# Decoupled contrastive multi-view clustering with adaptive false negative elimination for cancer subtyping

**DOI:** 10.1371/journal.pcbi.1013780

**Published:** 2025-12-04

**Authors:** Mengxiang Lin, Rongqi Fan, Saisai Zhu, Xiaoqiang Yan, Quan Zou, Zhen Tian

**Affiliations:** 1 School of Computer Science and Artificial Intelligence, Zhengzhou University, Zhengzhou, China; 2 Yangtze Delta Region Institute (Quzhou), University of Electronic Science and Technology of China, Quzhou, China; University of Pittsburgh, UNITED STATES OF AMERICA

## Abstract

Cancer’s heterogeneity necessitates precise subtype identification for effective diagnosis and treatment, which can be achieved by integrating multi-omics data to reveal distinct molecular characteristics and enable personalized therapies. Recently, significant efforts have been made through contrastive clustering methods to efficiently identify cancer subtypes. However, existing approaches remain limited in effectively capturing inter- and intra-view relationships in multi-omics data. Additionally, most cancer subtyping methods often rely on random sampling to construct negative pairs, which may inadvertently engender false negatives. To overcome these challenges, we propose a novel end-to-end self-supervised learning model named Decoupled Contrastive Multi-view Clustering with adaptive false negative elimination (DCMC). Specifically, DCMC adopts a multi-view clustering architecture that facilitates intra- and inter-view contrastive learning across distinct embedding spaces, allowing view-specific information to be preserved while maintaining cross-view consistency. We further introduce an adaptive false negative elimination framework to progressively screen potential false negatives. Finally, pseudo-label rectification is applied to enhance the quality of the learned representations and further refine the clustering process. DCMC is evaluated on 10 commonly used cancer datasets against 19 state-of-the-art methods, with experimental results validating its superior performance. In the Liver Hepatocellular Carcinoma case study, differential expression analysis is performed to identify potential biomarkers, while the cancer subtypes identified by DCMC are validated for their responses to specific therapeutic drugs. The datasets and source code for DCMC are available online at https://github.com/LinMengX/DCMC.

## Author summary

Cancer is a heterogeneous disease characterized by diverse etiologies and clinical features. Different cancer subtypes exhibit substantial variations in prognostic responses and treatment outcomes. Therefore, accurate subtype identification is crucial for effective diagnosis and personalized therapeutic interventions. However, existing methods for cancer subtyping struggle to capture the inter- and intra-view relationships in multi-omics data and often overlook the issue of false negatives in contrastive learning. To address these issues, we introduce DCMC, an end-to-end self-supervised learning model that employs a decoupled multi-view clustering architecture to preserve view-specific information while ensuring consistency across different omics. DCMC further enhances clustering quality by integrating an adaptive false negative elimination framework to progressively filter out misleading negative pairs, along with pseudo-label rectification to refine the learned representations. Through tests on 10 widely adopted cancer datasets, the experimental results consistently demonstrate that DCMC outperforms 19 state-of-the-art methods in terms of −log10 *P*-values and the number of enriched clinical labels. A case study on Liver Hepatocellular Carcinoma confirms the clinical relevance and therapeutic drug sensitivity of DCMC-identified subtypes.

## Introduction

Cancer is a complex disease involving abnormal cell growth characterized by uncontrolled proliferation, invasion of surrounding tissues, and potential metastasis to other regions of the body [1]. Traditional diagnostic approaches relied heavily on the morphological examination of tumors, although tumors with similar histopathological features often display significant differences in clinical progression and treatment responses [2]. Modern advances have revealed that each cancer type can consist of multiple subtypes, each with distinct molecular characteristics and clinical significance [3]. The identification of these cancer subtypes has become pivotal in precision medicine, enabling targeted therapies and improving treatment outcomes for patients [4]. By stratifying patients into biologically distinct subgroups, accurate cancer subtyping contributes to a more personalized approach to cancer diagnosis, prognosis, and therapy [5].

Rapid advancements in high-throughput sequencing technologies and biotechnological innovations have made the acquisition of diverse omics data, such as genomics, transcriptomics, proteomics, and epigenomics, increasingly accessible [6]. Comprehensive integration of these multi-omics datasets allows researchers to gain a holistic understanding of the molecular mechanisms underlying cancer. Unlike single-omics analysis, which only provides a limited view of molecular changes, multi-omics integration can capture the interplay among various molecular layers [7], thereby bridging the gap between genotype and phenotype. These advancements have driven the development of new computational methods aimed at more effectively identifying cancer subtypes, enhancing our understanding of tumor heterogeneity and facilitating personalized medicine. Large-scale international initiatives, such as The Cancer Genome Atlas (TCGA) [8] and the International Cancer Genome Consortium (ICGC) [9], have provided unprecedented opportunities to explore the complexities of cancer through multi-omics data, creating new possibilities and challenges for cancer research.

In recent years, researchers have increasingly focused their attention on the integration, analysis, and interpretation of large-scale multi-omics data [10]. Despite the valuable insights that comprehensive analysis of multi-omics data can offer across different levels, the effective integration of consistent information from multiple omics remains a significant challenge. According to the sequence of integration and clustering, existing methods can be classified into three main categories [11]: early integration, late integration, and intermediate integration.

Early integration methods combine multi-omics data into a single matrix, applying clustering algorithms like K-means [12] or Spectral clustering [13]. For example, LRAcluster [14] employs a low-rank approximation-based probabilistic model to integrate multi-omics data, yet it struggles with increased dimensionality and fails to account for differences in data distributions across omics. Late integration methods address some shortcomings of early integration by clustering each omics dataset independently and subsequently merging the clustering results. This approach, exemplified by methods such as CC [15] and PINSPLUS [16], provides greater robustness against noise and bias. Specifically, each omics dataset is clustered using the most suitable algorithm, and the resulting clusters are merged into a final consensus. However, both early and late integration methods fail to explicitly model interactions between omics layers, leading to the loss of crucial information from each independent omics clustering.

Intermediate integration methods jointly reduce dimensionality and cluster data by constructing a unified representation of multi-omics data without simple concatenation or independent clustering. Consequently, these methods have gradually become mainstream and can be further categorized into statistical, similarity-based, and deep learning-based methods [32]. MCCA [18] and MultiNMF [19] aim to maximize the correlation among multiple omics by projecting them into a lower-dimensional space. Meanwhile, iClusterBayes [20] employs Bayesian variable selection to model multi-omics data as latent variables, which helps in capturing the inherent structure of the data. Despite these advancements, traditional statistical methods continue to encounter challenges in accurately modeling complex and high-dimensional multi-omics data. Instead, similarity-based methods, such as SNF [21] and NEMO [24], introduce strategies to construct similarity matrices for each omics type and fuse them to capture inter-omics correlations. Specifically, SNF constructs a similarity network for each omics type and uses message passing to integrate these networks into a unified representation. SNFCC [22] combines SNF with CC to enhance clustering robustness, while NEMO applies radial basis function kernels for similarity calculation. Moreover, MSNE [23] leverages a random walk algorithm across multiple networks to integrate sample similarity, subsequently projecting the samples into a low-dimensional space. Although these methods improve inter-omics integration, they often rely on predefined similarity measures that may not always capture the intricate relationships among different data types.

More recently, deep learning-based methods have gained momentum for multi-omics cancer subtyping, leveraging the powerful feature extraction capabilities of neural networks. Subtype-GAN [25] is a deep adversarial learning model that extracts robust latent representations via adversarial training to tackle the heterogeneity of multi-omics data and employs consensus clustering with a Gaussian mixture model to identify distinct cancer subtypes. DSIR [26] and DLSF [27] both utilize deep subspace learning to derive a self-representation coefficient matrix, with the former integrating sparse subspace and manifold learning to capture global and local structures, and the latter employing a cycle autoencoder with a self-expressive layer to adaptively fuse nonlinear features. MRGCN [28] encodes omics-specific expression and reconstructs their similarity relationships through graph convolutional networks, consolidating full and partial multi-omics data into a unified latent embedding space. Harnessing a suite of independent variational autoencoders, DILCR [30] disentangles noise from omics data while capturing consistent latent representations.

Additionally, contrastive multi-view clustering (MvC) methods have emerged as another promising avenue for cancer subtype identification. In general, most existing MvC methods for cancer subtype identification usually use the off-the-shelf instances as positive pairs and construct negative pairs using random sampling [31]. For instance, Subtype-DCC [32] employs pseudo-labels generated by data augmentations, treating augmented samples from the same sample as positive pairs and other samples as negative pairs. Similarly, DMCL [33] leverages the multi-view settings, considering the same sample across different views as positive pairs and different instances, regardless of the view, as negative pairs. However, a common limitation of these approaches is their reliance on random sampling to construct negative pairs, which can inadvertently introduce false negatives (FNs) due to the misclassification of similar instances as negatives [34], thus misleading model optimization and degrading performance.

**Fig 1:**
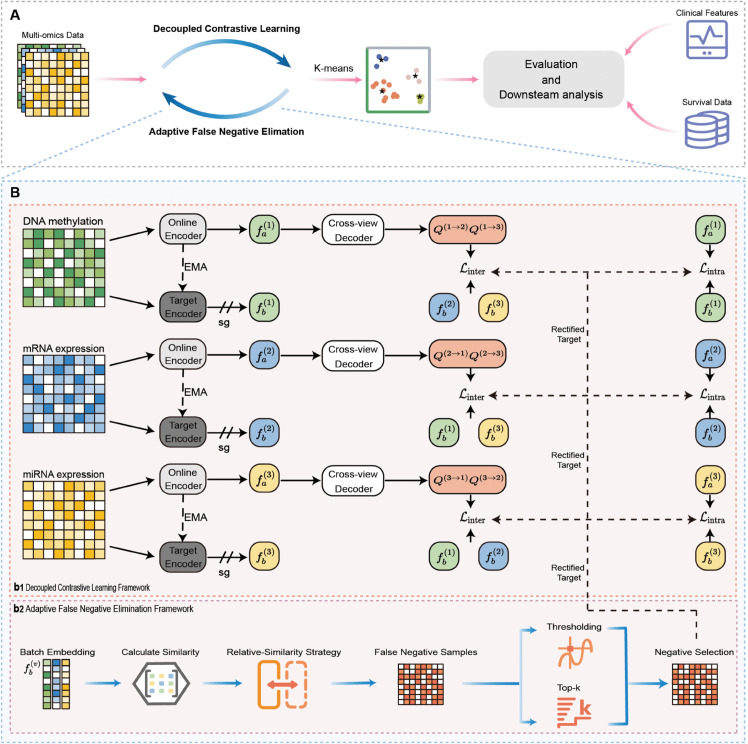
The overview of the DCMC model. **A** DCMC is an end-to-end self-supervised learning model for cancer subtyping using decoupled contrastive learning with adaptive false negative elimination. The multi-omics data are utilized as input features for the model. Once the embeddings are learned, K-means clustering is applied to identify cancer subtype clusters. Finally, the subtyping results undergo model evaluation and downstream analyses, which incorporate clinical features and survival data. **B(b1)** For each type of omics data, the dual embeddings $f_{a}^{\;(v)}$ and $f_{b}^{\;(v)}$ are generated by the view-specific Siamese encoder. The target encoder is updated via Exponential Moving Average (EMA), and sg denotes the stop-gradient operation. Subsequently, the cross-view decoder projects the embedding $f_{a}^{\;(v)}$ onto a latent representation space. **B(b2)** The similarity scores are calculated from each batch of embeddings $f_{b}^{\;(v)}$ and a relative-similarity strategy is applied to screen potential false negatives. To separate potential false negative samples, thresholding and top-*k* matching are utilized for negative selection. After that, DCMC leverages these selected negatives to rectify the targets of the overall loss function, thus effectively alleviating the adverse effects caused by false negatives.

Although the above methods have made significant advances in cancer subtyping, several unresolved challenges persist. Firstly, many approaches typically generate a unified latent representation by aggregating separately learned view-specific embeddings, which results in an inability to fully capture the genuine shared information across both inter- and intra-view relationships. Secondly, most existing contrastive methods rely on randomly selecting samples to construct negative pairs, which often leads to intra-cluster samples being incorrectly treated as negative pairs with a high probability. To address the above problems, we propose a method called Decoupled Contrastive Multi-view Clustering with adaptive false negative elimination (DCMC), as illustrated in Fig 1. Specifically, DCMC leverages a decoupled multi-view contrastive learning architecture that performs intra-view and inter-view contrastive learning in distinct feature spaces with the aid of a cross-view decoder, thereby preserving view-specific information while ensuring cross-view consistency. In addition, an adaptive false negative elimination framework is employed to screen and rectify potential false negatives, effectively reducing negative sampling bias and enhancing clustering performance. Comparative evaluations of DCMC and 19 alternative methods are performed on ten different multi-omics datasets, with experimental results consistently confirming that DCMC exhibits superior performance relative to other approaches. We further analyze the LIHC dataset to demonstrate the clinical relevance and the therapeutic drug sensitivity of the identified subtypes.

## Materials and methods

### Method overview

In this section, we provide a detailed description of the proposed method, namely Decoupled Contrastive Multi-view Clustering with adaptive false negative elimination for cancer subtyping (DCMC). As illustrated in Fig 1, the model comprises two primary modules: Decoupled Contrastive Learning Framework and Adaptive False Negative Elimination Framework. Specifically, we first utilize a decoupled contrastive learning framework to simultaneously capture cross-view consistency and preserve intra-view information. Moreover, we propose an adaptive framework to address potential false negatives by incorporating adaptive weights based on the similarity between the anchor and potential false negative samples. Finally, the multi-omics data undergo feature extraction through DCMC, followed by clustering to predict cancer subtypes, the identification of which is validated through comprehensive result analysis integrating clinical features and survival data. The implementation details of DCMC are elaborated upon in the following sections.

### Benchmark datasets

In this study, all methods are evaluated using ten cancer datasets from The Cancer Genome Atlas (TCGA), comprising multi-omics data for Acute Myeloid Leukemia (AML), Breast Invasive Carcinoma (BRCA), Colon Adenocarcinoma (COAD), Glioblastoma Multiforme (GBM), Kidney Renal Clear Cell Carcinoma (KIRC), Liver Hepatocellular Carcinoma (LIHC), Lung Squamous Cell Carcinoma (LUSC), Ovarian Serous Cystadenocarcinoma (OV), Sarcoma (SARC), and Skin Cutaneous Melanoma (SKCM). For each cancer type, integrative analyses are performed on three types of omics data, including DNA methylation, mRNA expression, and miRNA expression. Both multi-omics data and corresponding clinical information are directly obtained from reference [11]. A brief summary of these datasets is provided in S1 Table.

#### Generic multi-view datasets.

To validate the effectiveness of the proposed DCMC method, we conduct comprehensive experiments on four publicly available multi-view datasets. We begin with the COIL-20 dataset [35], a widely-used benchmark containing 1,440 object images categorized into 20 classes, where each image is represented through three heterogeneous feature types. The Fashion dataset [36] consists of 10,000 grayscale images of apparel from 10 categories, where three distinct feature sets are extracted from each image to form the views. Furthermore, the MSRC-v1 dataset [37] contains 210 images distributed across 7 distinct scene categories. For our experiments, we utilize three feature representations as separate views: CENT, CMT, and GIST. Finally, the Scene-15 dataset [38] includes 4,485 images from 15 scene classes, for which we adopt a three-view representation based on PHOG, GIST, and LBP features.

#### Data processing.

In the context of cancer datasets, features derived from RNA-seq and miRNA-seq data undergo logarithmic transformation, with subsequent exclusion of miRNA features exhibiting zero variance. Furthermore, the top 2,000 features exhibiting the highest variance are selected from both gene expression and DNA methylation data. All selected features are normalized to achieve a mean of zero and a standard deviation of one. Detailed descriptions of the processed cancer datasets are presented in Table 1, while the original datasets are provided in S2 Table. Additionally, to evaluate the model’s performance on a large and heterogeneous cohort, we construct a pan-cancer dataset [33] by integrating eight cancer datasets, in which the cancer type of origin serves as the ground-truth label for each sample. Comprehensive descriptions of the pan-cancer dataset are summarized in S1 Text.

**Table 1:** Comprehensive details for each cancer dataset employed in this study are provided. The datasets are characterized by the feature dimensions of mRNA expression, miRNA expression, and DNA methylation, respectively.

Datasets	Samples	mRNA expression	miRNA expression	DNA methylation
AML	170	2000	558	2000
BRCA	621	2000	891	2000
COAD	220	2000	613	2000
GBM	274	2000	534	2000
KIRC	183	2000	796	2000
LIHC	367	2000	852	2000
LUSC	341	2000	878	2000
OV	287	2000	616	2000
SARC	257	2000	838	2000
SKCM	448	2000	901	2000

### View-specific siamese encoders

Let $X=\{X^{1},X^{2},\ldots ,X^{V}\}$ denote a multi-omics dataset, where *V* represents the number of views. For the *v*-th omics measurement (v=1,2,…,V), $X^{\;(v)}=\{x_{1}^{v},x_{2}^{v},\ldots ,x_{N}^{v}{\}}^{T}\in {\mathbb{R}}^{D_{v}\times N}$ represents a collection of *N* data samples, each with a dimensionality of $D_{v}$. For each omics data type, the Siamese encoder [39] comprises an online encoder $g_{a}^{\;(v)}$ and a target encoder $g_{b}^{\;(v)}$, both sharing the same architecture but maintaining separate sets of weights. More specifically, the target encoder $g_{b}^{\;(v)}$ serves as a momentum encoder [40], with its weights $\theta_{b}$ updated as an Exponential Moving Average (EMA) of the online encoder’s parameters $\theta_{a}$:

$$\theta_{b}\leftarrow \xi \theta_{b}+(1-\xi )\theta_{a}$$ (1)

where $\theta_{b}$ and $\theta_{a}$ denote the parameters of the target encoder $g_{b}^{\;(v)}$ and the online encoder $g_{a}^{\;(v)}$, respectively. Here, ξ∈[0,1) is a momentum coefficient that controls the update rate of the target encoder. Only the parameters $\theta_{a}$ are updated through back-propagation, while the momentum update in Eq 1 ensures that $\theta_{b}$ evolves more smoothly than $\theta_{a}$, stabilizing the learning process.

For a given mini-batch of instances, the inputs are processed through $g_{a}^{\;(v)}$ and $g_{b}^{\;(v)}$ to generate the corresponding view-specific embeddings for each omics data type, defined as:

$$f_{a,i}^{\;(v)}=g_{a}^{\;(v)}(x_{i}^{v})$$ (2)

$$f_{b,i}^{\;(v)}=g_{b}^{\;(v)}(x_{i}^{v})$$ (3)

where $f_{a,i}^{\;(v)}$ represents the *i*-th output embedding of the sample $x_{i}^{v}$ processed by the online encoder $g_{a}^{\;(v)}$, while $f_{b,i}^{\;(v)}$ represents the *i*-th output embedding of the same sample $x_{i}^{v}$ processed by the target encoder $g_{b}^{\;(v)}$ in the *v*-th omics data type.

### Cross-view decoders

Although the aforementioned online and target encoders effectively capture intrinsic information from individual omics data types, their capacity to extract the beneficial shared information across different views is not guaranteed. Therefore, we introduce a cross-view decoder within the decoupled contrastive learning framework, which preserves view-specific information while simultaneously capturing cross-view consistency. Given the extracted embeddings from different omics data types, we aim to transfer cross-view information while preserving view-specific details to highlight the unique characteristics of each omics layer. Specifically, for a sample $x_{i}^{\;(v)}$, the online view embedding $f_{a,i}^{\;(v)}$ is projected into the embedding space corresponding to another view *k* via the cross-view decoder $p^{\left(v\to k\right)}$, resulting in the target embedding ${𝐐}_{i}^{\left(v\to k\right)}$, i.e.,

$${𝐐}_{i}^{\left(v\to k\right)}=p^{\left(v\to k\right)}f_{a,i}^{\;(v)}$$ (4)

The proposed cross-view decoders provide our framework with two significant advantages. Firstly, instead of relying on a single common space like existing methods, consistency learning is carried out in two separate view-specific spaces. This approach integrates the internal features of each view to highlight cross-view representations while preserving structural and complementary information unique to each view, which significantly enhances the diversity across different views and improves the performance of multi-view clustering (MvC) as verified in Table 4. Secondly, the cross-view decoders reconstruct samples from different perspectives [41], promoting feature integration and enhancing the interpretability of the learned representations. As a result, the model maintains robust cross-view consistency even with incomplete data, as demonstrated by the stable clustering performance at a 50% missing rate (Fig 4D–4G).

### Adaptive false negative elimination

A feasible approach to alleviate the impact of FNs is to identify potential false negatives within the set of negative pairs and then update the pseudo-target accordingly. With this in mind, several state-of-the-art contrastive MvC methods [42] seek to expand the set of positive samples in order to reduce the likelihood of introducing FNs. In particular, such approaches often employ *ϵ*-neighborhoods or *k*-nearest neighbors to refine the definition of positive relationships among instances [43]. However, relying solely on a basic neighborhood-based paradigm may result in under- and over-rectification. Specifically, samples belonging to the same cluster yet lying outside the neighborhood may still be misclassified as negatives, whereas inter-cluster samples within the neighborhood may be mistakenly labeled as positives [44].

To tackle the challenge of FNs in contrastive representation learning, we propose a robust framework designed to counteract negative sampling bias. More concretely, a relative-similarity strategy is first employed to identify potential FNs within the set of negative samples. Subsequently, we propose an Adaptive False Negative Elimination (AFNE) method to mitigate the impact of false negative samples detected during training. Furthermore, adaptive weights are computed based on similarity metrics to counteract performance degradation that arises from mistakenly assigning pseudo labels to certain identified false negatives. Fig 1 depicts the overall structural framework of AFNE.

#### False negative identification.

However, identifying FNs from the set of negative samples remains inherently challenging. To address this, a screening method known as the relative-similarity strategy is proposed. The approach designates a negative sample as a potential false negative based on the criterion that its similarity to the anchor closely approximates the similarity between the anchor and its corresponding positive sample.

We utilize two common screening criteria to separate potential FNs from the set of negative samples: thresholding and top-*k* matching. The top-*k* strategy is preferred when the approximate number of FNs is required, while thresholding is more suitable when dynamic adjustment is expected. During initial training iterations, representations generated by the online and target encoders often lead to many samples satisfying the thresholding criterion. To this end, we integrate thresholding for dynamic adjustments with top-*k* matching, which limits the number of FNs to a predefined maximum *k*, thereby improving the reliability of false negative identification. The detailed steps for applying the relative-similarity strategy to identify potential FNs are outlined below:

1. For each anchor *i* (i=1,2,…,N), extract its embedding representation $f_{b,i}^{\;(v)}$ from the Target Encoder, and generate the positive sample’s representation ${\hat{f}}_{b,i}^{\;(v)}$. Subsequently, retrieve the representations $f_{b,j}^{\;(v)}$ (j≠i,j=1,2,…,N) of negative samples *j* (j≠i,j=1,2,…,N).
2. Obtain the similarity scores between the anchor and each negative sample, $S_{i,j}^{\;(v)}=\text{sim}(f_{b,i},f_{b,j})$, along with the similarity score between the anchor and its corresponding positive sample, $S_{i,i^{+}}^{\;(v)}=\text{sim}(f_{b,i},{\hat{f}}_{b,i})$, where *i*^ + ^ denotes the positive counterpart to anchor *i*.
3. Compute the relative similarity scores between the anchor-positive similarity and each anchor-negative similarity $R_{i,j}^{\;(v)}=\left|S_{i,i^{+}}^{\;(v)}-S_{i,j}^{\;(v)}\right|$, (j≠i,j=1,2,…,N). Negative samples with small relative similarity scores ($R_{i,j}^{\;(v)}$) are likely to be false negatives.
4. Define the set of potential false negatives ${\mathbb{N}}_{i}$ as the negative samples that exhibit the highest similarity to the anchor, satisfying the condition ${\mathbb{N}}_{i}=\{j|0<R_{i,j}^{\;(v)}<t\text{ and }R_{i,j}^{\;(v)}\in \text{top}(R_{i,j}^{\;(v)},k)\}$, where *t* represents the threshold value, *k* denotes the number of assigned potential false negatives, and top(·,·) denotes the set of *k* negative samples with the smallest relative similarity scores.

#### Adaptive weighting for false negative elimination.

Actually, AFNE builds upon the traditional false negative elimination (FNE) [45] by incorporating adaptive weights. False negative elimination [45] is a widely used approach for addressing identified false negative samples in contrastive learning. While the conventional practice involves removing identified false negatives from the set of negative samples, AFNE adopts an alternative strategy by retaining these samples and assigning them adaptive weights. Our false negative detection strategy employs the target encoder for representation extraction. Due to the target encoder potentially being undertrained during the initial training iterations, the resulting representations may lack reliability. Consequently, the initially selected false negatives from the screening process could comprise true negative samples. As demonstrated in Table 5, directly applying FNE to handle the identified FNs can give rise to performance degradation.

To mitigate this problem, we introduce an adaptive weighting mechanism that dynamically adjusts the weights between the identified FNs and the anchor. The matrix $\Lambda \in {\mathbb{R}}^{N\times N}$ serves as the adaptive weight matrix, with its elements $\alpha_{i,m}$ defined as follows:

$$\alpha_{i,m}=\left\{ \begin{array}{cc} 1-\frac{\exp(sim(f_{b,i}^{\;(v)},{\hat{f}}_{b,m}^{\;(v)}))}{\sum_{\begin{array}{c} j=1,j\neq i \end{array}}^{N}\exp(sim(f_{b,i}^{\;(v)},f_{b,j}^{\;(v)}))} & \text{if }m\in {\mathbb{N}}_{i}\\ 1 & \text{if }m\notin {\mathbb{N}}_{i} \end{array}\right.$$ (5)

where sim(⋅,⋅) denotes cosine similarity, ${\hat{f}}_{b,m}^{\;(v)}$ denotes the representation of the detected false negative, and ${\mathbb{N}}_{i}$ represents the set of identified FNs.

The adaptive weight functions as a confidence measure, quantifying the likelihood that a putative false negative is truly a positive sample. Samples showing low cosine similarity to the anchor receive higher weights, as they are more likely to be true negatives rather than false negatives. Conversely, high-similarity samples are likely genuine false negatives and thus assigned lower weights. All remaining negative samples outside of ${\mathbb{N}}_{i}$ are assigned a full weight of 1 to retain their contribution. After obtaining the adaptive weights, the pseudo target matrix ${𝐓}^{\;(v)}\in {\mathbb{R}}^{N\times N}$ is finally computed as follows:

$${𝐓}^{\;(v)}=\Lambda ⊙{𝐒}^{\;(v)}$$ (6)

where Λ is the adaptive weight matrix that adjusts the contribution of each similarity score based on the likelihood of being a false negative, and ${𝐒}^{\;(v)}$ is the similarity matrix containing pairwise similarities between the anchor and each negative sample.

### Decoupled contrastive loss

As described above, our framework employs distinct contrastive objectives to enhance both intra-view discrimination and inter-view consistency, achieved by an encoder–decoder architecture that transforms data from a shared space into separate view-specific representations. After obtaining the pseudo-target matrices ${𝐓}^{\;(v)}$, the intra-view contrastive loss is defined as:

$${ℒ}_{\text{intra}}=\sum_{v=1}^{V}W({𝐓}^{\;(v)},\rho (f_{a}^{\;(v)},f_{b}^{\;(v)}))$$ (7)

where W(·,·) denotes the cross entropy, and the function ρ(·,·) is the pairwise similarity with the row-wise normalization operation:

$$[\rho (\gamma ,\delta )]ij=\frac{\exp(s(\gamma_{i},\delta_{j})/\tau )}{\sum_{l=1}^{n}\exp(s(\gamma_{i},\delta_{l})/\tau )}$$ (8)

where τ denotes the temperature, which is fixed at 0.5 across all experimental configurations, and s(·,·) represents the similarity function. For intra-view contrastive learning, ${𝐓}^{\;(v)}$ is employed to improve the discrimination of embeddings within the same view by dynamically adjusting the contribution of false negatives. Conversely, to enhance cross-view consistency, a cross-view target ${𝐓}^{\left(k\right)}$ is constructed based on the similarity structure derived from the latent representations of the predicted view, boosting cross-view interactions and achieving better cross-view consistency. Therefore, we can formalize the inter-view contrastive loss as:

$${ℒ}_{\text{inter}}=\sum_{v\neq k}^{V}W({𝐓}^{\left(k\right)},\rho ({𝐐}^{\left(v\to k\right)},f_{b}^{\left(k\right)}))$$ (9)

Finally, the overall decoupled contrastive loss is then given by:

$$ℒ={ℒ}_{\text{intra}}+{ℒ}_{\text{inter}}$$ (10)

In summary, ${ℒ}_{\text{intra}}$ enhances intra-view discrimination by dynamically adjusting the contribution of false negatives based on view-specific representations, while ${ℒ}_{\text{inter}}$ promotes cross-view consistency by aligning representations across different views. The complete approach is outlined in Algorithm 1.

**Algorithm 1 DCMC.**

**Input:** Multi-omics datasets: $X=\{X^{1},X^{2},\ldots ,X^{V}\}$; Target encoder parameters: $\theta_{b}$

**Output:** Final cluster labels: *c*

1: For each omics data type *X^(v)^*, pass the input through both the online encoder to obtain the embedding $f_{a,i}^{\;(v)}$ and the target encoder to generate the embedding $f_{b,i}^{\;(v)}$ according to Eq 2 and Eq 3;

2: The cross-view decoder projects the embedding $f_{a,i}^{\;(v)}$ into latent representation space corresponding to another view k, resulting in the target embedding by (Eq 4);

3: Apply the relative-similarity strategy to screen out candidate false negatives;

4: Calculate the adaptive weights by (Eq 5);

5: Update pseudo target matrix ${𝐓}^{\;(v)}$ via (Eq 6);

6: Compute the intra-view contrastive loss ${ℒ}_{\text{intra}}$ and the inter-view contrastive loss ${ℒ}_{\text{inter}}$ using Eq 7 and Eq 9, respectively;

7: Backpropagate loss and update target encoder parameters $\theta_{b}$ based on (Eq 1);

8: Extract the final features for each omics data type using both target encoders and cross-view decoders;

9: Perform K-means clustering to obtain the final cluster labels c.

### Complexity analysis

Let *N*, *V*, *D*, *B*, and *E* denote the number of samples, omics views, embedding dimensionality, batch size, and the number of training epochs, respectively. The complexities of the view-specific Siamese encoders and the cross-view decoders are both $𝒪(NVD^{2})$ per epoch, assuming fully connected layers where input and output dimensions are on the order of *D*. The adaptive false negative elimination (AFNE) framework and the inter-view contrastive loss, which are dominated by pairwise similarity computations quadratic in the batch size, both contribute a complexity of 𝒪(NB) per epoch. The exponential moving average (EMA) update of target encoder parameters is comparatively negligible. Therefore, the overall time complexity per epoch is $𝒪(NVD^{2}+NB)$, and the complexity for the entire training process becomes $𝒪(E(NVD^{2}+NB))$.

## Results

### Experimental settings

Our framework is developed in Python 3.9.16 using PyTorch 1.13.0 and runs on a Windows 11 system with NVIDIA GeForce RTX 3080 GPUs. The model is trained for 5 independent runs (200 epochs per run) with a fixed batch size of 256, enabling efficient optimization across runs while maintaining consistent performance across epochs. During the training process, a warmup strategy is implemented over the initial 20 epochs to progressively increase the learning rate prior to its stabilization. The view-specific encoder employs a four-layer fully connected network (FCN) with batch normalization and ReLU activation, while the cross-view decoder utilizes a two-layer MLP containing an expanded hidden layer followed by ReLU non-linearity. For the remaining hyperparameters, the contrastive temperature *τ* is maintained at 0.5, the threshold is fixed at 0.7, and the top-*k* parameter is determined to be 3 throughout the experiments.

### Performance evaluation

#### Comparison approaches and evaluation metrics.

We conduct comprehensive experiments on ten benchmark datasets to evaluate DCMC’s clustering performance in cancer subtyping, comparing it with 19 state-of-the-art methods for multi-omics integration. These approaches comprise early integration methods, consisting of K-means [12], Spectral [13], and LRAcluster [14]; late integration methods, involving CC [15] and PINSPlus [16]; and 14 intermediate integration methods. For the intermediate integration methods, one kernel learning method, three statistical methods, three similarity-based methods, and seven deep learning-based methods are employed: rMKL-LPP [17], MCCA [18], MultiNMF [19], iClusterBayes [20], SNF [21], SNFCC [22], MSNE [23], NEMO [24], DLSF [27], DSIR [26], MRGCN [28], MOCSS [29], DMCL [33], and DILCR [30]. Among them, DMCL and DILCR utilize contrastive learning approaches to enhance cancer subtype identification. All competing methods are implemented using the default configurations provided by their respective authors.

Due to the absence of well-defined cancer subtypes in the ten multi-omics datasets [54], we assess the performance of each cancer subtyping method using two widely adopted evaluation metrics. First, the log-rank test [47] is employed to calculate the −log10 *P*-values from survival analysis, which determines whether significant differences exist among cancer subtypes identified by DCMC. Second, we evaluate the clinical relevance of the identified clusters through clinical label enrichment analysis. Specifically, six clinical parameters are selected for enrichment testing: age at diagnosis, gender, pathologic T, pathologic M, pathologic N, and pathologic stage. Among these, the latter four serve as discrete pathological metrics that quantify tumor progression (T), metastases (M), lymph node involvement (N), and overall cancer progression (pathologic stage). For statistical evaluation, the Chi-square test is applied to the discrete clinical labels, while the Kruskal–Wallis test is utilized for continuous clinical labels. In addition, not all cancer datasets include all six aforementioned clinical labels, and the specific clinical labels employed in each dataset are provided in S3 Table.

#### Performance evaluation by DCMC on cancer datasets.

The performance comparison in Table 2 demonstrates DCMC’s superiority relative to 19 integration methods across 10 cancer datasets, with evaluations based on survival analysis −log10 *P*-values and clinical label enrichment counts. It is clearly evident from the results that the clusters identified by DCMC demonstrate significant survival differences across all 10 cancer datasets. To be more specific, our proposed method outperforms the other 19 methods by achieving higher −log10 *P*-values in survival analysis on all datasets, indicating that the cancer subtypes identified by DCMC exhibit more pronounced differences. While DCMC has fewer significant clinical parameters than SNF and MSNE on the COAD dataset, and shows comparable performance to several methods on LIHC, it consistently achieves higher −log10 *P*-values than all competing approaches across datasets.

**Table 2:** Performance comparison of DCMC against 19 integration methods across all datasets. Each cell presents the results in the format A/B(C), where *A* represents enriched clinical labels detected, *B* denotes the −log10 *P*-values obtained from survival analysis, and *C* indicates the number of clusters. Statistical significance is defined as *P*-values < 0.05, with significant outcomes highlighted in bold. Means represent the algorithm’s average value, while Sig denotes the number of datasets that yield significant results.

Methods	AML	BRCA	COAD	GBM	KIRC	LIHC	LUSC	OV	SARC	SKCM	Means	Sig
K-means	1/**3.2**(5)	1/0.6(2)	1/0.1(2)	2/**2.6**(5)	4/**1.4**(4)	2/0.2(2)	1/0.2(2)	1/0.1(2)	2/**1.3**(2)	3/**2.4**(5)	1.8/1.2	10/5
Spectral	1/**1.8**(9)	2/**1.8**(3)	1/0.2(2)	2/**2.5**(5)	5/**1.9**(4)	2/0.4(2)	2/0.3(2)	1/0.8(4)	2/**1.3**(2)	2/**1.7**(5)	2.0/**1.3**	10/6
LRAcluster	1/**2.9**(7)	4/**1.6**(7)	1/0.1(5)	2/**2.4**(11)	2/0.6(3)	2/**4.0**(12)	1/0.2(5)	1/0.2(9)	2/**2.5**(5)	3/1.2(15)	1.9/**1.6**	10/5
CC	1/**3.8**(3)	3/**2.8**(5)	1/0.3(2)	2/**2.8**(7)	4/**1.4**(7)	3/**3.0**(3)	1/0.4(4)	1/0.2(4)	2/**2.7**(4)	1/**3.0**(5)	1.9/**2.0**	10/7
PINSPLUS	1/**1.6**(4)	2/**1.4**(5)	1/0.1(4)	1/**3.0**(2)	3/**1.7**(6)	2/0.5(5)	2/0.4(2)	1/0.1(2)	2/1.1(3)	1/**1.7**(3)	1.6/1.2	10/5
SNF	1/**3.1**(4)	2/1.0(2)	3/0.3(2)	1/**4.1**(2)	5/**2.7**(3)	2/0.1(2)	1/0.6(3)	1/0.1(2)	2/**1.8**(3)	2/1.0(3)	2.0/**1.5**	10/4
SNFCC	1/**2.8**(4)	2/**1.5**(5)	2/0.1(10)	2/**3.8**(9)	5/**3.6**(2)	2/1.2(10)	1/0.5(2)	1/0.4(3)	2/**1.8**(3)	2/**4.4**(5)	2.0/**2.0**	10/6
rMKL-LPP	1/**2.8**(6)	4/0.6(7)	2/0.5(6)	2/**3.4**(6)	4/**1.3**(11)	4/1.0(6)	1/0.3(6)	1/0.1(6)	2/**2.5**(6)	2/**2.6**(7)	2.3/**1.5**	10/5
MCCA	2/**1.8**(11)	2/**4.8**(14)	2/0.3(2)	2/**2.1**(11)	2/**1.8**(15)	2/**1.3**(15)	2/0.2(12)	1/0.8(9)	2/0.8(15)	2/**4.4**(2)	1.9/**1.8**	10/6
MultiNMF	1/**1.3**(2)	1/**1.3**(2)	1/0.4(2)	1/**2.2**(3)	4/**2.0**(2)	3/**3.0**(3)	2/0.3(2)	1/0.3(2)	2/1.1(2)	2/**5.1**(2)	1.8/**1.7**	10/6
iClusterBayes	1/**2.2**(5)	2/**2.2**(5)	1/0.9(5)	2/**2.5**(5)	4/**1.5**(5)	2/**2.4**(5)	1/0.1(5)	1/0.1(5)	2/**4.0**(5)	4/**5.6**(5)	2.0/**2.2**	10/7
NEMO	1/**3.5**(5)	3/**2.0**(4)	1/0.6(2)	2/**3.0**(10)	5/**2.7**(3)	4/**4.2**(5)	1/0.5(3)	1/0.4(3)	2/**1.9**(3)	2/**4.8**(10)	2.2/**2.4**	10/7
DLSF	1/**2.5**(5)	2/**1.9**(3)	1/0.1(4)	2/**4.5**(5)	3/**2.8**(4)	3/**3.3**(3)	1/0.1(3)	1/0.3(4)	2/**2.4**(10)	3/**3.9**(5)	1.9/**2.2**	10/7
MSNE	1/**1.8**(5)	1/**2.5**(5)	3/**1.7**(5)	1/**3.0**(2)	2/**1.5**(4)	3/1.2(5)	1/0.2(2)	1/0.6(3)	1/**3.0**(5)	2/**2.0**(4)	1.6/**1.8**	10/7
DSIR	1/**2.7**(7)	3/**6.8**(12)	1/**1.1**(5)	2/**3.0**(9)	4/**1.4**(4)	2/**2.0**(10)	2/**1.8**(3)	1/1.0(3)	2/**2.6**(3)	3/**3.7**(8)	2.1/**2.6**	10/8
MRGCN	1/**3.0**(10)	4/**6.7**(4)	1/0.6(7)	2/**3.8**(8)	4/**2.4**(9)	2/**1.7**(10)	1/**1.5**(13)	1/0.8(5)	2/**3.3**(8)	3/**4.5**(5)	2.1/**2.9**	10/8
MOCSS	1/**3.5**(4)	3/**2.8**(5)	2/0.8(5)	2/**5.2**(3)	4/**4.0**(4)	2/0.7(3)	3/0.4(5)	1/0.6(3)	2/**2.2**(4)	3/**5.0**(5)	2.3/**2.5**	10/6
DMCL	1/**2.1**(9)	4/**2.1**(3)	1/0.1(2)	2/0.1(4)	5/**1.7**(6)	2/**2.6**(4)	1/0.2(4)	1/0.7(5)	2/**3.2**(6)	2/**2.3**(5)	2.1/**1.5**	10/6
DILCR	1/**5.5**(5)	3/**3.1**(5)	1/0.8(3)	2/**2.5**(5)	5/**1.6**(4)	2/**2.9**(4)	1/0.6(3)	1/0.6(2)	2/**2.1**(5)	1/1.1(5)	1.9/**2.1**	10/6
DCMC(ours)	1/**7.0**(3)	4/**8.1**(5)	2/**2.9**(4)	2/**7.1**(5)	5/**7.2**(4)	3/**9.4**(5)	1/**3.3**(3)	1/**3.2**(5)	2/**9.2**(5)	3/**9.8**(5)	2.4/**6.7**	10/10

**Fig 2:**
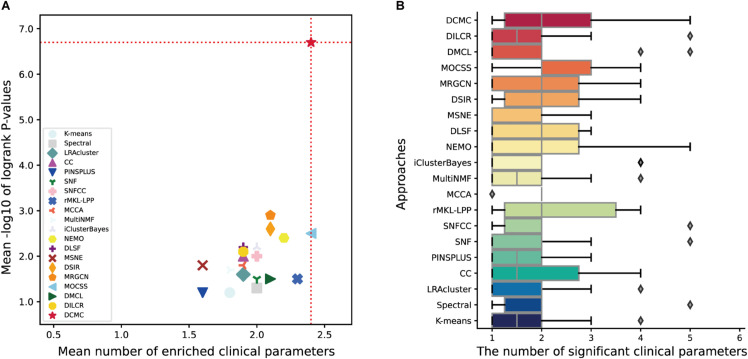
Evaluation of DCMC against alternative approaches on 10 cancer datasets. A The mean performance of the various integration methods. The X-axis represents the average number of enriched clinical parameters in the clusters and the Y-axis represents the average −log10 P-values. The red dotted line indicates the best performance achieved among all methods. B Comparative assessment of the significant clinical parameters identified by DCMC and alternative approaches. The X-axis lists the clustering methods evaluated, while the Y-axis represents the number of significant clinical parameters.

As shown in Fig 2A, DCMC achieves higher average −log10 *P*-values across the 10 datasets compared to all alternative methods, and the average number of significant clinical labels is the same as that of MOCSS. As illustrated in Fig 2B, for the enrichment analysis of clinical labels, DCMC exhibits performance that either surpasses or is comparable to that of the other comparison approaches. To determine the subtypes in each cancer, we follow configurations reported in previous studies and consider 3, 4, and 5 as candidate cluster numbers. The detailed comparison results for all three clustering configurations are provided in S4 Table. As can be seen from Table 2, the determined number of subtypes for each cancer dataset is indicated in parentheses. Furthermore, the enriched clinical parameters for all comparison methods are summarized in S5 Table.

**Fig 3:**
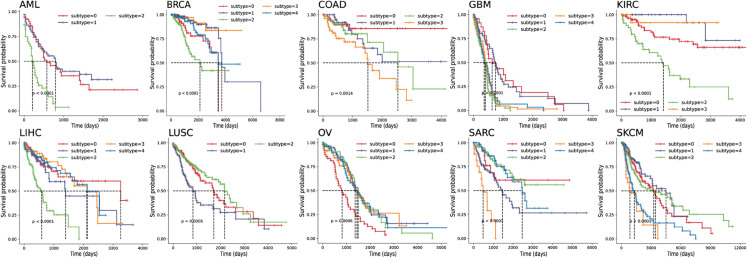
Kaplan-Meier survival curves of DCMC across all multi-omics datasets. Different cancer subtypes are depicted by uniquely colored curves, while the median survival time is indicated by a dashed line. The extent to which the curves diverge illustrates the significance of survival differences among patients belonging to various subtypes. The black dashed line typically represents the baseline survival curve for the overall patient cohort, serving as a reference point against which the survival outcomes of the identified subtypes are compared.

The Kaplan-Meier survival analysis in Fig 3 demonstrates DCMC’s ability to effectively stratify patients into distinct prognostic groups across all 10 cancer datasets, with clearly separated survival curves for each identified subtype. For instance, in the KIRC dataset, the identified cancer subtypes exhibit markedly different survival curves. Notably, subtype_3 demonstrates a higher survival rate compared to the other subtypes around 3000 days. This distinct separation underscores the clinical relevance of the identified subtypes and supports the potential of DCMC in guiding personalized treatment strategies.

**Fig 4:**
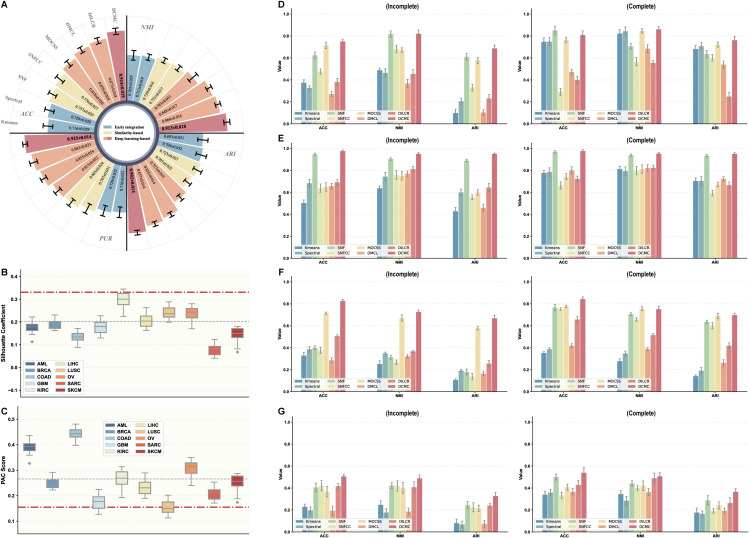
Comprehensive evaluation of DCMC on pan-cancer and multi-view datasets. **A** Clustering performance on the integrated pan-cancer dataset measured by ACC, NMI, ARI, and purity metrics. **B,C,** Clustering quality assessment using the silhouette coefficient (higher is better) and PAC score (lower is better) on both original and processed data. The red dot-dashed line represents the performance obtained by DCMC on preprocessed datasets, while the gray dotted line indicates its performance on original high-dimensional datasets. **D–G,** Results on four benchmark multi-view datasets under complete and incomplete view settings: COIL-20 (D), Fashion (E), MSRC-v1 (F), and Scene-15 (G).

To further evaluate the robustness and scalability of DCMC on large-scale and heterogeneous multi-omics data, we conduct a comprehensive assessment on an integrated pan-cancer dataset, where the cancer type of origin serves as the ground-truth label. We compare DCMC against competitive methods representing early integration (Spectral, K-means), similarity-based (SNF, SNFCC), and deep learning-based (DMCL, MOCSS, DILCR) approaches. As shown in Fig 4A, clustering performance is evaluated using four standard metrics: Accuracy (ACC) [48], Normalized Mutual Information (NMI) [49], Adjusted Rand Index (ARI) [50], and purity [51]. For these metrics, a larger value indicates better clustering performance. Our proposed DCMC method consistently achieves superior performance, outperforming all baseline methods across all four metrics. For example, compared to the second-best method, DCMC achieves performance improvements of approximately 5.84%, 5.43%, 5.25%, and 4.76% in terms of the ACC, NMI, ARI, and purity metrics, respectively. These results highlight DCMC’s strong ability to identify meaningful clusters within complex and large-scale multi-omics datasets.

#### Comprehensive comparison on multi-view datasets.

To validate the effectiveness and robustness of our proposed method, we conduct comprehensive experiments on four widely-used multi-view datasets. Our model is benchmarked against several baseline methods under both complete and incomplete data conditions. The complete setting assumes all views are present, while the more challenging incomplete setting involves a 50% rate of samples with missing views. The performance is evaluated using three standard metrics: ACC, NMI, and ARI.

Under the complete setting, where all view information is available, our method consistently achieves superior and highly competitive performance across all four datasets (Fig 4D–4G). For instance, on the COIL-20 (Fig 4D) and Fashion (Fig 4E) datasets, our model surpasses all baseline methods across the three evaluation metrics. Furthermore, our model demonstrates notable robustness under the more challenging incomplete setting. While the performance of most baseline methods degrades significantly with a 50% view missing rate, our model demonstrates remarkable resilience and robust clustering performance. We attribute this exceptional stability to the efficacy of our cross-view decoders. By reconstructing data from available views, the decoders ensure the model learns robust cross-view consistency, effectively mitigating the negative impact of incomplete data. This is especially clear on the MSRC-v1 (Fig 4F) and Scene-15 (Fig 4G) datasets, where our method shows only a minor decrease in performance compared to the substantial drop experienced by other methods.

#### Scalability evaluation of DCMC.

While the pan-cancer experiments demonstrate DCMC’s excellent performance on large-sample datasets, we conduct a comprehensive evaluation on ten unprocessed cancer datasets to confirm its effectiveness on high-dimensional data. These raw datasets contain significantly more features (e.g., up to 20,531 for mRNA expression) and include missing omics data. In addition to survival analysis and clinical label enrichment, we introduce two metrics to assess clustering stability: the silhouette coefficient [52] and the proportion of ambiguous clustering (PAC) [53]. The silhouette coefficient quantifies clustering validity by assessing for each sample whether it is more similar to members of its own cluster than to members of other clusters [54]. The PAC score assesses clustering stability by measuring the proportion of ambiguously clustered sample pairs in a consensus matrix derived from subsampling, where a lower value indicates a more consistent clustering structure [55].

As depicted in Fig 4B and 4C, our model achieves strong clustering quality on both raw and preprocessed data. While the average silhouette coefficient and PAC scores are marginally better with preprocessed data, the competitive performance on the original data demonstrates the model’s robustness. A similar conclusion can be drawn from the survival and clinical enrichment outcomes (S1 Fig), where the model obtains robust −log10 *P*-values and enriched clinical labels directly from the raw data, even though these metrics are further improved by preprocessing. As shown in the runtime analysis (S2 Fig), training on the original datasets is substantially more time-consuming than on their preprocessed counterparts. This increased computational cost is a direct consequence of the high dimensionality of the original datasets. Overall, the model’s ability to effectively handle such large-scale and high-dimensional multi-omics datasets underscores its scalability.

The unprocessed datasets used in our evaluation contain inherent missing views, with detailed sample counts provided in S6 Table. Our model architecture is designed to handle this challenge through its cross-view decoders. To process a sample with a missing view *k*, we leverage an observed view *v* to recover its representation via the decoder $p^{\left(v\to k\right)}$, formulated as ${𝐐}_{i}^{\left(v\to k\right)}=p^{\left(v\to k\right)}f_{b,i}^{\;(v)}$, where $f_{b,i}^{\;(v)}$ is the embedding from the observed view. The effectiveness of this mechanism is demonstrated by DCMC’s strong performance on both general multi-view datasets with missing views (Fig 4D–4G) and the multi-omics cancer datasets (Fig 4B–4C). Notably, the COAD dataset, which exhibits the highest average missing rate, shows no significant performance degradation across all metrics, demonstrating the model’s robustness in handling missing views.

Finally, we investigate the impact of our feature selection strategy, which retains the top 2,000 most variable features. We compare this against three alternatives: selecting the top 3,000 most variable features, selecting 3,000 random features, and using the original unprocessed high-dimensional data. The results in S7 Table show that selecting the top 2,000 features consistently provides the best performance in survival analysis. In contrast, using 3,000 randomly selected features leads to a significant drop in performance. Although selecting the top 3,000 features occasionally achieves competitive performance (e.g., on GBM with a −log10 *P*-values of 7.4 compared to 7.1), it generally underperforms the top 2,000 selection and shows less stable clustering quality as evidenced by lower silhouette scores and higher PAC scores on most datasets. This confirms that selecting features based on high variance is an effective strategy that focuses the model on biologically relevant signals and is superior to both random selection and the inclusion of less informative, potentially noisy features.

### Ablation studies

In this section, we conduct a series of ablation studies to demonstrate the validity and contribution of DCMC’s key components, primarily using the GBM, KIRC, and LIHC datasets. To be more specific, we first examine the impact of individual components and further explore various modifications of the decoupled contrastive learning paradigm. We then provide a detailed justification for the cross-view decoder architecture by comparing view-specific versus shared decoders and analyzing its sensitivity to depth and capacity. Finally, we quantify the significant contribution of the Adaptive False Negative Elimination (AFNE) module, demonstrating both its effectiveness within DCMC and its generalizability as a plug-and-play component for other methods.

#### Ablation analysis of model components.

As can be seen from Table 3, we sequentially isolate each component and evaluate its performance based on −log10 *P*-values from survival analysis. In the absence of the cross-view decoder, ${ℒ}_{\text{inter}}$ is applied directly to the representations *f*_*a*_ and *f*_*b*_. Additionally, it is noteworthy that in the ablation study of the false negative rectification strategy, we directly remove both the pseudo-target matrix and the identity matrix. The experimental results demonstrate the varying contributions of individual components to the overall performance. Specifically, the cross-view decoder achieves a 9% improvement. Moreover, retaining both ${ℒ}_{\text{intra}}$ and ${ℒ}_{\text{inter}}$ together improves the average results by 14% compared to applying them separately. In brief, each component of DCMC is indispensable to its overall effectiveness.

**Table 3:** Ablation study of our method on GBM, KIRC, and LIHC, where ✓ denotes the components adopted and ✗ denotes the components removed.

${ℒ}_{\text{intra}}$	${ℒ}_{\text{inter}}$	Decoder	Rectification	GBM	KIRC	LIHC
				-log10 P-values		
✓	✗	✗	✗	6.0	4.5	5.1
✗	✓	✗	✗	5.5	3.6	6.0
✓	✓	✗	✗	6.1	5.0	6.4
✓	✓	✓	✗	6.6	5.6	6.8
✓	✓	✗	✓	6.9	6.0	7.2
✓	✓	✓	✓	7.1	7.2	9.4

#### Ablation analysis of the decoupled contrastive learning framework.

To better understand the design of the decoupled contrastive learning framework, we analyze the impact of two critical mechanisms: momentum-based encoder updating and the application of the stop-gradient operation. In this context, the “share” setting replaces the target encoder with the online encoder, eliminating the momentum update. When the decoder is removed, we perform the same procedures as described above. Additionally, the stop-gradient operation prevents gradient flow to the target encoder during backpropagation, while its parameters are updated through the EMA of the online encoder weights. As presented in Table 4, removing either mechanism leads to a marked decline in performance, underscoring its essential role in maintaining distinct view-specific representations.

**Table 4:** Ablation study of momentum updating strategy, stop-gradient operation, and cross-view decoder.

Target Encoder	Stop Gradient	Decoder	GBM	KIRC	LIHC
			-log10 P-values		
Share	✗	✗	5.5	4.1	6.2
Share	✓	✗	5.7	4.4	5.2
Momentum	✓	✗	5.9	5.1	7.3
Share	✗	✓	5.8	4.7	7.0
Share	✓	✓	6.1	5.2	8.3
Momentum	✓	✓	7.1	7.2	9.4

#### Ablation analysis of cross-view decoders.

The design of the cross-view decoders is justified by two additional ablation studies that address the use of view-specific versus shared decoders and the sensitivity of the model to decoder architecture.

First, we evaluate whether using view-specific decoders offers an advantage over a single shared decoder. As can be seen from S8 Table, the use of specific decoders consistently and substantially outperforms a shared decoder architecture across all ten cancer datasets, evidenced by higher −log10 *P*-values and more enriched clinical labels. For instance, on the BRCA and SARC datasets, the −log10 *P*-values improve from 4.4 and 6.3 to 8.1 and 9.2, respectively. Furthermore, higher silhouette scores and lower PAC scores on nearly all datasets demonstrate that the view-specific decoders significantly improve cluster quality. This result confirms that modeling the unique relationships between different omics modalities with specific decoders is crucial for performance.

Second, we investigate the sensitivity of DCMC to the decoder’s architectural depth and capacity using the AML and LIHC datasets. The decoder depth is set to 3, 4, and 5 layers, while its hidden dimension is scaled by a width multiplier of 0.5 and 2.0 relative to the default setting to evaluate the impact of architectural complexity. As summarized in S9 Table, the default configuration consistently outperforms the alternatives across all evaluation metrics. In addition, we observe that both simplifying and increasing the complexity of the decoder relative to our default configuration lead to a notable decline in performance, yet several second-best results are obtained when the depth is set to five layers. Notably, the model proves to be more sensitive to changes in the decoder’s width than its depth.

#### Ablation analysis of AFNE.

Adaptive False Negative Elimination (AFNE) is built upon the FNE method by introducing adaptive weights that improve its ability to discern potential false negatives from negative pairs. Several experiments are conducted to demonstrate that adding adaptive weights helps enhance performance. In contrast to the ablation study of the false negative rectification strategy, here we use the identity matrix *I* as the pseudo-target matrix. Superior performance of AFNE over both FNE and baseline implementations is demonstrated in Table 5. The standard FNE method provides a consistent improvement over the baseline, enhancing performance by an average of 13% across the three datasets. In comparison, our proposed AFNE method offers a far more substantial gain, outperforming the baseline by an average of 44%. Collectively, the results from our ablation studies illustrate that among all tested components, the AFNE method provides the most significant contribution to the model’s overall performance.

**Table 5:** Comparison of DCMC performance with and without AFNE and FNE across three datasets.

Strategy	GBM	KIRC	LIHC
	-log10 P-values		
W/O AFNE	5.6	6.0	5.1
FNE	5.9	6.2	6.7
AFNE	7.1	7.2	9.4

Furthermore, to demonstrate the generalizability and effectiveness of our proposed AFNE module, we integrate it into several competitive baseline methods, including DLSF, MOCSS, DMCL, and DILCR. As summarized in S10 Table, incorporating AFNE consistently enhances performance across the ten TCGA datasets, as evidenced by higher survival analysis −log10 *P*-values and, in some cases, an increased number of enriched clinical labels. Specifically, the integration of the AFNE module improves the mean −log10 *P*-values for DLSF, MOCSS, DMCL, and DILCR from 2.2, 2.5, 1.5, and 2.1 to 2.4, 3.0, 2.0, and 3.0, respectively. Taken together, these results demonstrate that AFNE serves as a versatile and effective plug-and-play module, capable of boosting the performance of diverse multi-view clustering methods on complex cancer datasets.

### Single-view and multi-view data comparative evaluation

**Fig 5:**
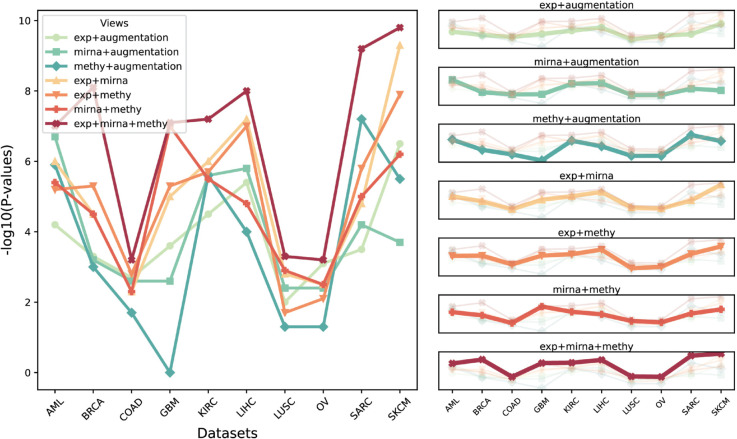
Comparison of −log10 *P*-values across cancer types for different view combinations. Left: The X-axis indicates the ten cancer datasets used for comparison and the Y-axis indicates −log10 *P*-values. Right: The bold lines indicate the view combinations for the current subfigure.

To verify that leveraging multi-view information effectively contributes to the model’s performance, experiments are carried out across datasets under different view combinations. In particular, for datasets containing only a single view, data augmentation is adopted to generate paired samples suitable for decoupled contrastive learning. We specifically employ complex Gaussian noise as the augmentation strategy to generate varied representations [56]. The noise intensity is dynamically adjusted based on the feature range of the data, with the base standard deviation set to 5% of the feature range. Furthermore, non-uniform noise is applied, where the standard deviation varies across dimensions, ensuring diversity in the augmented data. The performance comparison of our method across ten commonly utilized cancer datasets is presented in Fig 5, with more detailed results available in S11 Table. It is evident that clustering performance is enhanced as additional views are integrated, and the proposed method consistently outperforms single-view approaches on multi-view data.

### Runtime and memory usage analysis

In this section, we analyze the computational performance of DCMC against baseline methods, with detailed results presented in S12 Table and S13 Table. To ensure a comprehensive comparison, the selected baselines represent three major categories of multi-omics integration strategies: early, late, and intermediate integration. All experiments are conducted under the same hardware and software environment. The reported runtime is the average of five independent runs, and the memory usage reflects the maximum memory allocated during execution.

As shown in S12 Table, DCMC demonstrates a moderate and efficient memory footprint. Its memory consumption is highly competitive with other deep learning-based (intermediate integration) methods. For example, on the BRCA dataset, DCMC requires 325 MB of memory, a figure comparable to DMCL (378 MB) and notably lower than DILCR (402 MB). More significantly, DCMC exhibits a clear efficiency advantage over classical early-integration approaches such as LRAcluster (18,103 MB) and similarity-based intermediate-integration methods like SNFCC (37,320 MB), both of which demand substantially higher memory usage.

In terms of runtime, the results in S13 Table indicate that DCMC is more computationally intensive than the baseline methods. For instance, on the BRCA dataset, DCMC’s runtime is 23,840 seconds, which is considerably longer than other deep learning models, including DMCL (170 seconds) and DILCR (214 seconds). This increased computational overhead is primarily attributed to its decoupled learning architecture and the iterative nature of the Adaptive False Negative Elimination (AFNE) module, which performs complex similarity screenings within each batch. The substantial gains in clustering accuracy, survival analysis significance, and clinical relevance, as demonstrated in Table 2, justify the additional computational investment.

### Parameter sensitivity analysis

**Fig 6:**
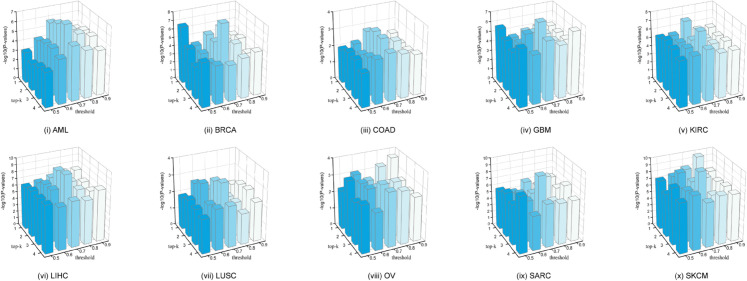
Comparison of the −log10 *P*-values across different parameter settings of threshold and top-*k*. The threshold values are defined as {0.5,0.6,0.7,0.8,0.9}, while the top-*k* parameter is set to {1,2,3,4}. A grid search is utilized to assess the performance under varying combinations of threshold and top-*k*. The X-axis indicates the threshold values, the Y-axis denotes the top-*k* parameter, and the Z-axis represents −log10 of the log-rank test *P*-values.

Our parameter sensitivity analysis focuses on three key parameters: temperature *τ*, thresholding, and top-*k*. The temperature parameter (*τ*) controls the sharpness of the similarity distribution. To reveal its impact, we vary *τ* from 0 to 1 and assess how it influences the −log10 *P*-values across ten cancer datasets. As demonstrated in S3 Fig, although this parameter has certain impacts on model performance, setting τ=0.5 generally yields comparable and reliable results. Two other important parameters, thresholding and top-*k*, are used to balance the dynamic adjustment of false negative criteria and ensure the number of false negatives remains within a predefined limit, respectively. Furthermore, we individually investigate the effect of these two hyperparameters on the performance of DCMC. The threshold is assigned values from {0.5,0.6,0.7,0.8,0.9} and *k* is chosen from {1,2,3,4}. A grid search is employed to evaluate performance across various combinations of threshold and *k*. The experimental results for the ten cancer datasets are presented in Fig 6, while more detailed results are recorded in S14 Table. We observe that although the effects of the two parameters vary across the ten cancer datasets, setting the threshold to 0.7 and top-*k* at 3 tends to achieve better results.

### Case study

As depicted in Table 2, DCMC exhibits robust performance on the LIHC dataset, as evidenced by its high −log10 *P*-values in survival analysis and the significant number of enriched clinical labels. The Kaplan-Meier survival curves (Fig 3) clearly separate the survival outcomes of the identified subtypes, underscoring DCMC’s capability to capture meaningful clinical stratification. This separation further demonstrates the model’s effectiveness in delineating subtypes with distinct prognostic implications and emphasizes its clinical relevance in subtype classification and survival prediction.

To further evaluate the clinical relevance of DCMC, we perform a systematic characterization of the five distinct cancer subtypes it identified in the LIHC dataset. To this end, we first conduct differential expression analysis (DEA) to identify subtype-specific biomarkers by performing t-tests on each feature across different omics data and ranking them accordingly [57]. Subsequently, Gene Ontology (GO) and KEGG enrichment analyses are performed to gain insights into the molecular pathways and biological processes associated with these differentially expressed genes. Finally, we assess differential drug sensitivity among molecularly defined liver cancer subtypes.

We first perform a t-test (P-adjust≤0.05, FoldChange>1) to identify genes with significant differential expression in mRNA across LIHC subtypes, selecting the most significant genes based on the *P*-value. The t-SNE visualization of these subtypes, presented in S4 Fig, illustrates distinct spatial distributions among them. Moreover, the visualization of subtype-specific biomarkers for each subtype reveals that differentially expressed genes (DEGs) provide an intuitive separation among the subtypes (S5 Fig). Specifically, the most prominent biomarkers show relatively high expression levels within their respective clusters (highlighted in red) and comparatively low expression in other clusters (highlighted in blue). Taken together, this emphasizes a robust association between the selected biomarkers and their corresponding molecular subtypes.

**Fig 7:**
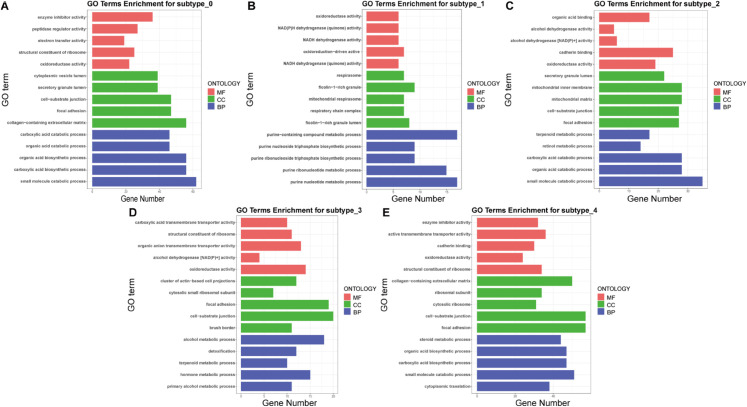
Top 5 significantly enriched GO terms for each of the following categories: molecular functions (MF), cellular components (CC), and biological processes (BP). **A–E** display the enrichment analysis results of differentially expressed genes for subtype_0 through subtype_4, respectively.

**Fig 8:**
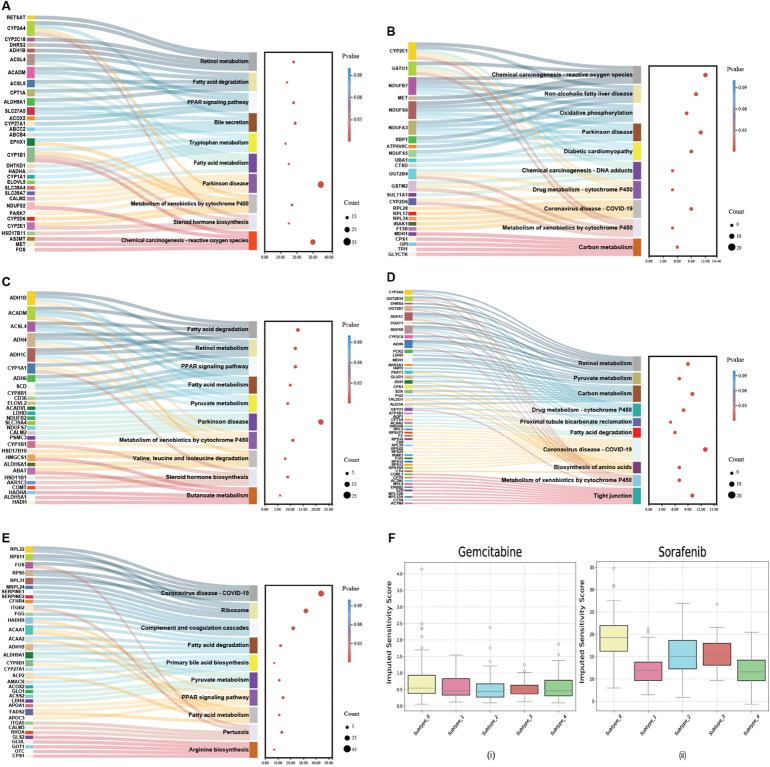
Top enriched KEGG pathways of each set of differentially expressed genes in LIHC and associated variations in therapeutic drug efficacy across liver cancer subgroups. **A** Key KEGG pathway for LIHC subtype_0. **B** Key KEGG pathway for LIHC subtype_1. **C** Key KEGG pathway for LIHC subtype_2. **D** Key KEGG pathway for LIHC subtype_3. **E** Key KEGG pathway for LIHC subtype_4. **F** The imputed drug sensitivity scores for (i) Gemcitabine and (ii) Sorafenib.

To elucidate the biological significance of identified biomarkers, we conduct GO enrichment analysis and KEGG pathway enrichment analysis on DEGs using the ‘clusterProfiler‘ R package [60]. Fig 7 demonstrates significant enrichment of DEGs in key GO terms, while Fig 8 displays their KEGG pathway enrichment. The KEGG pathway enrichment analysis results reveal the significant pathways enriched with differentially expressed genes (Fig 8A, 8B, 8C, 8D, 8E). Overall, these enriched pathways shed light on pivotal biological processes and metabolic adaptations that may contribute to hepatocellular carcinoma progression, suggesting potential therapeutic or diagnostic targets for further investigation.

Notably, subtype-specific analyses further delineate metabolic divergence within these pathways, with distinct enrichment patterns observed across molecular subgroups. For subtype_0, differentially expressed genes are predominantly enriched in processes related to small molecule catabolism and carboxylic acid biosynthesis (Fig 7A). In subtype_1, these genes are mainly involved in purine nucleotide metabolism (Fig 7B), while in subtype_2 they are chiefly associated with small molecule catabolic processes (Fig 7C). The gene set for subtype_3 is primarily enriched in primary alcohol metabolism and hormone metabolism (Fig 7D), and for subtype_4, the differentially expressed genes are mainly linked to cytoplasmic translation (Fig 7E). Collectively, these LIHC-associated biological processes strengthen the clinical relevance of the biomarkers by anchoring them to disease-specific molecular mechanisms.

We further analyze the variations in therapeutic drug efficacy across different liver cancer subtypes [61]. Specifically, we apply the R package ‘oncoPredict‘ [62] to estimate the therapeutic sensitivity indices for two widely utilized liver cancer treatments, Sorafenib and Gemcitabine. Sorafenib is a multikinase inhibitor that chiefly acts by inhibiting various enzymes involved in promoting tumor growth and angiogenesis [63]. Gemcitabine is a deoxycytidine analog that interferes with the replication process of cancer cells by mimicking DNA components, thereby suppressing their proliferation [64]. In particular, we train the prediction model using datasets from the Sanger Genomics of Drug Sensitivity in Cancer (GDSC) v2 database and subsequently calculate therapeutic sensitivity scores. The Kruskal–Wallis test is applied to quantify the statistical differences in the imputed scores among the five subtypes. As depicted in Fig 8F, for Sorafenib, subtype_0 exhibits greater sensitivity, whereas subtype_1 and subtype_4 show lower sensitivity compared to the other subtypes. Conversely, Gemcitabine demonstrates generally lower estimated scores than Sorafenib, indicating that patients are more likely to respond effectively to Gemcitabine. Taken together, the distinct biological characteristics of each subtype dictate their sensitivity or resistance to specific therapeutic drugs, underscoring the importance of tailoring chemotherapeutic treatments to each patient’s subtype.

## Discussion

In this study, we propose a robust decoupled contrastive learning framework that incorporates adaptive false negative elimination for application in the cancer subtyping task. Specifically, DCMC leverages decoupled contrastive learning to effectively integrate multi-omics data, preserving view-specific information while maintaining cross-view consistency. Furthermore, we incorporate an adaptive false negative elimination strategy that employs dynamic weighting based on relative similarity to mitigate the adverse effects of misclassified negatives in contrastive learning, ultimately enhancing the robustness of clustering outcomes. As demonstrated by the experimental results, DCMC exhibits superior performance compared to 19 current alternatives on 10 extensively utilized multi-omics datasets. More in-depth experiments on the LIHC dataset underscore the promising potential of DCMC for clinical applications. The visualization of mRNA expression levels for the most prominent biomarkers identified by DCMC clearly demonstrates that the cancer subtypes possess distinct biological significance. Enrichment analysis of signaling pathways based on the differentially expressed genes aligns with findings from previous studies, thereby supporting the predictive and therapeutic value of the identified subtypes. Additionally, we analyze therapeutic drug efficacy across liver cancer subtypes using drug sensitivity scores estimated with the ’oncoPredict’ R package, revealing distinct drug responses among subtypes. Overall, we expect that identifying distinct cancer subtypes with the proposed method contributes to improved clinical outcome predictions, provides valuable insights into cancer development and treatment, and accelerates advancements in personalized therapy.

Although DCMC demonstrates superior performance on the majority of TCGA datasets, there undoubtedly remains room for further refinement. For instance, one important direction for future work is to incorporate protein expression data and integrate protein–protein interactions to enhance the interpretability of the integrative embedding. Additionally, while false negative elimination mitigates the negative impact of comparing with false negatives, it also overlooks the valuable information present in true positives. In future research, identified false negatives could be treated as true positives, and the anchor points could be drawn toward the positive samples, thereby improving overall model performance. Resolving these challenges in subsequent work will drive the continued expansion and refinement of DCMC.

## Supporting information

S1 Table The detailed information of the ten benchmark TCGA datasets evaluated in this work. (PDF)

S2 Table Summary of the high-dimensional original multi-omics datasets. (PDF)

S3 Table Clinical labels selected for each cancer dataset. ✓ denotes that the dataset includes the clinical labels, whereas × represents that the clinical labels are absent.
(PDF)

S4 TableDCMC is evaluated across ten cancer datasets using multiple clustering configurations (3, 4, and 5 clusters).The **bold numbers** in the table denotes the optimal number of clusters selected for each dataset. In the *A*/*B* format, *A* represents the −log10 *P*-values of survival analysis, while *B* indicates the number of enriched clinical parameters.(PDF)

S5 Table The significant clinical parameters enriched by different methods. (PDF)

S6 Table Summary of missing omics data across ten TCGA cancer datasets. During preprocessing, we exclude non-tumor samples and remove records with missing or duplicate entries. The average missing rate represents the proportion of samples with at least one missing omics modality among all retained samples.
(PDF)

S7 Table Comparison of clustering and survival analysis performance under different feature selection strategies across ten TCGA cancer datasets. (PDF)

S8 Table Comparison between shared and specific decoders across ten cancer datasets. (PDF)

S9 Table Sensitivity analysis of decoder architecture on AML and LIHC datasets. Note: The best results in each dataset are shown in **bold** face, and • indicates the second-best result.
(PDF)

S10 TablePerformance comparison of competitive methods with and without Adaptive False Negative Elimination (AFNE) across ten TCGA cancer datasets.Each cell presents the results in the format A/B(C), where *A* denotes the number of enriched clinical labels, *B* is the −log10 *P*-values from survival analysis, and *C* indicates the number of clusters. Means represent the algorithm’s average value. **Bold values** highlights the superior results obtained by methods integrated with AFNE.(PDF)

S11 TableClustering performance of DCMC across the ten cancer datasets under different view combinations.In each cell, results are shown as *A*/*B*, where *A* represents the number of significant clinical labels and *B* denotes the −log10 *P*-values obtained from survival analysis.(PDF)

S12 Table Memory usage (MB) of DCMC and baseline methods across 10 TCGA datasets. (PDF)

S13 Table Run time comparisons of baseline methods on all datasets(in seconds). (PDF)

S14 Table Sensitivity analysis of threshold and top-k selection on survival significance across ten cancer datasets. (PDF)

S1 FigPerformance comparison between original and processed datasets in terms of survival significance (−log10 *P*-values) and the number of enriched clinical parameters.(TIFF)

S2 Fig Computational runtime comparison between original and processed data across ten TCGA datasets. (TIFF)

S3 FigImpacts of temperature *τ* on the clustering performance on the ten cancer datasets.We adjust this parameter within the range of 0 to 1 and evaluate its impact on the −log10 *P*-values across ten cancer datasets.(TIFF)

S4 Fig The t-SNE visualization of DCMC subtypes on the LIHC dataset. (TIFF)

S5 Fig The visualization of the most promising potential biomarkers identified by DCMC on the LIHC dataset. Note: Red represents high expression, whereas blue indicates low expression.
(TIFF)

S1 Text The description of pan-cancer dataset. (DOCX)

## References

[1] Tarin D. Cell and tissue interactions in carcinogenesis and metastasis and their clinical significance. Semin Cancer Biol. 2011;21(2):72–82. doi: 10.1016/j.semcancer.2010.12.006 21147229

[2] He L, Long LR, Antani S, Thoma GR. Histology image analysis for carcinoma detection and grading. Comput Methods Programs Biomed. 2012;107(3):538–56. doi: 10.1016/j.cmpb.2011.12.007 22436890 PMC3587978

[3] Dai X, Li T, Bai Z, Yang Y, Liu X, Zhan J, et al. Breast cancer intrinsic subtype classification, clinical use and future trends. Am J Cancer Res. 2015;5(10):2929–43. 26693050 PMC4656721

[4] Torres C, Grippo PJ. Pancreatic cancer subtypes: a roadmap for precision medicine. Ann Med. 2018;50(4):277–87. doi: 10.1080/07853890.2018.1453168 29537309 PMC6151873

[5] Tran KA, Kondrashova O, Bradley A, Williams ED, Pearson JV, Waddell N. Deep learning in cancer diagnosis, prognosis and treatment selection. Genome Med. 2021;13(1):152. doi: 10.1186/s13073-021-00968-x 34579788 PMC8477474

[6] Chakraborty S, Hosen MI, Ahmed M, Shekhar HU. Onco-multi-OMICS approach: a new frontier in cancer research. Biomed Res Int. 2018;2018:9836256. doi: 10.1155/2018/9836256 30402498 PMC6192166

[7] Leng D, Zheng L, Wen Y, Zhang Y, Wu L, Wang J, et al. A benchmark study of deep learning-based multi-omics data fusion methods for cancer. Genome Biol. 2022;23(1):171. doi: 10.1186/s13059-022-02739-2 35945544 PMC9361561

[8] Cancer Genome Atlas Research Network, Weinstein JN, Collisson EA, Mills GB, Shaw KRM, Ozenberger BA, et al. The cancer genome atlas pan-cancer analysis project. Nat Genet. 2013;45(10):1113–20. doi: 10.1038/ng.2764 24071849 PMC3919969

[9] Zhang J, Baran J, Cros A, Guberman JM, Haider S, Hsu J, et al. International cancer genome consortium data portal–a one-stop shop for cancer genomics data. Database (Oxford). 2011;2011:bar026. doi: 10.1093/database/bar026 21930502 PMC3263593

[10] Subramanian I, Verma S, Kumar S, Jere A, Anamika K. Multi-omics data integration, interpretation, and its application. Bioinform Biol Insights. 2020;14:1177932219899051. doi: 10.1177/1177932219899051 32076369 PMC7003173

[11] Rappoport N, Shamir R. Multi-omic and multi-view clustering algorithms: review and cancer benchmark. Nucleic Acids Res. 2018;46(20):10546–62. doi: 10.1093/nar/gky889 30295871 PMC6237755

[12] Hartigan JA, Wong MA. Algorithm AS 136: a K-means clustering algorithm. Applied Statistics. 1979;28(1):100. doi: 10.2307/2346830

[13] von Luxburg U. A tutorial on spectral clustering. Stat Comput. 2007;17(4):395–416.

[14] Wu D, Wang D, Zhang MQ, Gu J. Fast dimension reduction and integrative clustering of multi-omics data using low-rank approximation: application to cancer molecular classification. BMC Genomics. 2015;16:1022. doi: 10.1186/s12864-015-2223-8 26626453 PMC4667498

[15] Monti S, Tamayo P, Mesirov J, Golub T. Consensus clustering: a resampling-based method for class discovery and visualization of gene expression microarray data. Mach Learn. 2003;52:91–118.

[16] Nguyen H, Shrestha S, Draghici S, Nguyen T. PINSPlus: a tool for tumor subtype discovery in integrated genomic data. Bioinformatics. 2019;35(16):2843–6. doi: 10.1093/bioinformatics/bty1049 30590381

[17] Speicher NK, Pfeifer N. Integrating different data types by regularized unsupervised multiple kernel learning with application to cancer subtype discovery. Bioinformatics. 2015;31(12):i268-75. doi: 10.1093/bioinformatics/btv244 26072491 PMC4765854

[18] Witten DM, Tibshirani RJ. Extensions of sparse canonical correlation analysis with applications to genomic data. Stat Appl Genet Mol Biol. 2009;8(1):Article28. doi: 10.2202/1544-6115.1470 19572827 PMC2861323

[19] Liu J, Wang C, Gao J, Han J. Multi-view clustering via joint nonnegative matrix factorization. In: Proc SIAM Int Conf Data Min. 2013. p. 252–60.

[20] Mo Q, Shen R, Guo C, Vannucci M, Chan KS, Hilsenbeck SG. A fully Bayesian latent variable model for integrative clustering analysis of multi-type omics data. Biostatistics. 2018;19(1):71–86. doi: 10.1093/biostatistics/kxx017 28541380 PMC6455926

[21] Wang B, Mezlini AM, Demir F, Fiume M, Tu Z, Brudno M, et al. Similarity network fusion for aggregating data types on a genomic scale. Nat Methods. 2014;11(3):333–7. doi: 10.1038/nmeth.2810 24464287

[22] Xu T, Le TD, Liu L, Su N, Wang R, Sun B, et al. CancerSubtypes: an R/Bioconductor package for molecular cancer subtype identification, validation and visualization. Bioinformatics. 2017;33(19):3131–3. doi: 10.1093/bioinformatics/btx378 28605519

[23] Kunz T, Rieber L, Mahony S. Assessing relationships between chromatin interactions and regulatory genomic activities using the self-organizing map. Methods. 2021;189:12–21. doi: 10.1016/j.ymeth.2020.07.002 32652235

[24] Rappoport N, Shamir R. NEMO: cancer subtyping by integration of partial multi-omic data. Bioinformatics. 2019;35(18):3348–56. doi: 10.1093/bioinformatics/btz058 30698637 PMC6748715

[25] Imrie F, Bradley AR, Deane CM. Generating property-matched decoy molecules using deep learning. Bioinformatics. 2021;37(15):2134–41. doi: 10.1093/bioinformatics/btab080 33532838 PMC8352508

[26] Kirchler M, Konigorski S, Norden M, Meltendorf C, Kloft M, Schurmann C, et al. transferGWAS: GWAS of images using deep transfer learning. Bioinformatics. 2022;38(14):3621–8. doi: 10.1093/bioinformatics/btac369 35640976

[27] Zhang C, Chen Y, Zeng T, Zhang C, Chen L. Deep latent space fusion for adaptive representation of heterogeneous multi-omics data. Brief Bioinform. 2022;23(2):bbab600. doi: 10.1093/bib/bbab600 35079777

[28] Yang B, Yang Y, Wang M, Su X. MRGCN: cancer subtyping with multi-reconstruction graph convolutional network using full and partial multi-omics dataset. Bioinformatics. 2023;39(6):btad353. doi: 10.1093/bioinformatics/btad353 37255323 PMC10279523

[29] Chen Y, Wen Y, Xie C, Chen X, He S, Bo X, et al. MOCSS: Multi-omics data clustering and cancer subtyping via shared and specific representation learning. iScience. 2023;26(8):107378. doi: 10.1016/j.isci.2023.107378 37559907 PMC10407241

[30] Cai Y, Wang S. Deeply integrating latent consistent representations in high-noise multi-omics data for cancer subtyping. Brief Bioinform. 2024;25(2):bbae061. doi: 10.1093/bib/bbae061 38426322 PMC10939425

[31] Yang B, Cui C, Wang M, Ji H, Gao F. Multi-view multi-level contrastive graph convolutional network for cancer subtyping on multi-omics data. Brief Bioinform. 2024;26(1):bbaf043. doi: 10.1093/bib/bbaf043 39899598 PMC11789786

[32] Zhao J, Zhao B, Song X, Lyu C, Chen W, Xiong Y, et al. Subtype-DCC: decoupled contrastive clustering method for cancer subtype identification based on multi-omics data. Brief Bioinform. 2023;24(2):bbad025. doi: 10.1093/bib/bbad025 36702755

[33] Chen W, Wang H, Liang C. Deep multi-view contrastive learning for cancer subtype identification. Brief Bioinform. 2023;24(5):bbad282. doi: 10.1093/bib/bbad282 37539822

[34] Wang X, Yang S, Zhang J, Wang M, Zhang J, Yang W, et al. Transformer-based unsupervised contrastive learning for histopathological image classification. Med Image Anal. 2022;81:102559. doi: 10.1016/j.media.2022.102559 35952419

[35] Nene SA, Nayar SK, Murase H. Columbia Object Image Library (COIL-100). CUCS-006-96. Department of Comput Science, Columbia University; 1996.

[36] Xiao H, Rasul K, Vollgraf R. Fashion-MNIST: A novel image dataset for benchmarking machine learning algorithms. arXiv prerpint 2017. arXiv:1708.07747

[37] Winn J, Jojic N. LOCUS: learning object classes with unsupervised segmentation. In: Tenth IEEE International Conference on Computer Vision (ICCV’05) Volume 1. 2005. 756–763 Vol. 1. 10.1109/iccv.2005.148

[38] Fei-Fei Li, Perona P. A Bayesian hierarchical model for learning natural scene categories. In: 2005 IEEE Computer Society Conference on Computer Vision and Pattern Recognition (CVPR’05). p. 524–31. 10.1109/cvpr.2005.16

[39] Grill JB, Strub F, Altché F, Tallec C, Richemond P, Buchatskaya E. Bootstrap your own latent: a new approach to self-supervised learning. Adv Neural Inf Process Syst. 2020;33:21271–84.

[40] He K, Fan H, Wu Y, Xie S, Girshick R. Momentum contrast for unsupervised visual representation learning. In: Proceedings of the IEEE/CVF Conference on Computer Vision and Pattern Recognition. 2020. p. 9729–38.

[41] Shou Y, Lan H, Cao X. Contrastive graph representation learning with adversarial cross-view reconstruction and information bottleneck. Neural Netw. 2025;184:107094. doi: 10.1016/j.neunet.2024.107094 39799719

[42] Zhong H, Wu J, Chen C, Huang J, Deng M, Nie L, et al. Proceedings of the IEEE/CVF International Conference on Computer Vision. 2021. p. 9224–33.

[43] Yang M, Li Y, Hu P, Bai J, Lv J, Peng X. Robust multi-view clustering with incomplete information. IEEE Trans Pattern Anal Mach Intell. 2023;45(1):1055–69. doi: 10.1109/TPAMI.2022.3155499 35230947

[44] Robinson J, Chuang CY, Sra S, Jegelka S. Contrastive learning with hard negative samples. In: ICLR. 2021. p. 1–15.

[45] Huynh T, Kornblith S, Walter MR, Maire M, Khademi M. Boosting contrastive self-supervised learning with false negative cancellation. In: Proceedings of the IEEE/CVF Winter Conference on Applications of Computer Vision (WACV) 2022. p. 2785–95.

[46] Duan R, Gao L, Gao Y, Hu Y, Xu H, Huang M, et al. Evaluation and comparison of multi-omics data integration methods for cancer subtyping. PLoS Comput Biol. 2021;17(8):e1009224. doi: 10.1371/journal.pcbi.1009224 34383739 PMC8384175

[47] Mukhopadhyay P, Ye J, Anderson KM, Roychoudhury S, Rubin EH, Halabi S, et al. Log-Rank Test vs MaxCombo and difference in restricted mean survival time tests for comparing survival under nonproportional hazards in immuno-oncology trials: a systematic review and meta-analysis. JAMA Oncol. 2022;8(9):1294–300. doi: 10.1001/jamaoncol.2022.2666 35862037 PMC9305601

[48] Wu M, Schölkopf B. A local learning approach for clustering. Adv Neural Inf Process Syst. 2006;19:1529-36.

[49] Estévez PA, Tesmer M, Perez CA, Zurada JM. Normalized mutual information feature selection. IEEE Trans Neural Netw. 2009;20(2):189–201. doi: 10.1109/TNN.2008.2005601 19150792

[50] Hara K, Nakayama Y, Miyoshi S, Okada M. Mutual learning with many linear perceptrons: on-line learning theory. Lecture Notes in Computer Science. Berlin, Heidelberg: Springer; 2009. p. 171–80. 10.1007/978-3-642-04274-4_18

[51] Chen J, Mao H, Wang Z, Zhang X. Low-rank representation with adaptive dictionary learning for subspace clustering. Knowledge-Based Systems. 2021;223:107053. doi: 10.1016/j.knosys.2021.107053

[52] Rousseeuw PJ. Silhouettes: a graphical aid to the interpretation and validation of cluster analysis. Journal of Computational and Applied Mathematics. 1987;20:53–65. doi: 10.1016/0377-0427(87)90125-7

[53] Șenbabaoğlu Y, Michailidis G, Li JZ. Critical limitations of consensus clustering in class discovery. Sci Rep. 2014;4:6207. doi: 10.1038/srep06207 25158761 PMC4145288

[54] Duan R, Gao L, Gao Y, Hu Y, Xu H, Huang M, et al. Evaluation and comparison of multi-omics data integration methods for cancer subtyping. PLoS Comput Biol. 2021;17(8):e1009224. doi: 10.1371/journal.pcbi.1009224 34383739 PMC8384175

[55] John CR, Watson D, Russ D, Goldmann K, Ehrenstein M, Pitzalis C, et al. M3C: Monte Carlo reference-based consensus clustering. Sci Rep. 2020;10(1):1816. doi: 10.1038/s41598-020-58766-1 32020004 PMC7000518

[56] Ye Y, Li Y, Ouyang R, Zhang Z, Tang Y, Bai S. Improving machine learning based phase and hardness prediction of high-entropy alloys by using Gaussian noise augmented data. Comput Mater Sci. 2023;223:112140.

[57] He Y, Wang X. Identifying biomarkers associated with immunotherapy response in melanoma by multi-omics analysis. Comput Biol Med. 2023;167:107591. doi: 10.1016/j.compbiomed.2023.107591 37875043

[58] Gene Ontology Consortium. The Gene Ontology in 2010 : extensions and refinements. Nucleic Acids Res. 2010;38(Database issue):D331-5. doi: 10.1093/nar/gkp1018 19920128 PMC2808930

[59] Kanehisa M, Goto S. KEGG: Kyoto encyclopedia of genes and genomes. Nucleic Acids Res. 2000;28(1):27–30. doi: 10.1093/nar/28.1.27 10592173 PMC102409

[60] Yu G, Wang L-G, Han Y, He Q-Y. clusterProfiler: an R package for comparing biological themes among gene clusters. OMICS. 2012;16(5):284–7. doi: 10.1089/omi.2011.0118 22455463 PMC3339379

[61] Luo W, Xiang W, Gan L, Che J, Li J, Wang Y, et al. Bulk and single-cell transcriptome profiling reveal necroptosis-based molecular classification, tumor microenvironment infiltration characterization, and prognosis prediction in colorectal cancer. J Transl Med. 2022;20(1):235. doi: 10.1186/s12967-022-03431-6 35590418 PMC9118791

[62] Maeser D, Gruener RF, Huang RS. oncoPredict: an R package for predicting in vivo or cancer patient drug response and biomarkers from cell line screening data. Brief Bioinform. 2021;22(6):bbab260. doi: 10.1093/bib/bbab260 34260682 PMC8574972

[63] Wilhelm SM, Adnane L, Newell P, Villanueva A, Llovet JM, Lynch M. Preclinical overview of sorafenib, a multikinase inhibitor that targets both Raf and VEGF and PDGF receptor tyrosine kinase signaling. Mol Cancer Ther. 2008;7(10):3129–40. doi: 10.1158/1535-7163.MCT-08-0013 18852116 PMC12261297

[64] Mini E, Nobili S, Caciagli B, Landini I, Mazzei T. Cellular pharmacology of gemcitabine. Ann Oncol. 2006;17 Suppl 5:v7-12. doi: 10.1093/annonc/mdj941 16807468

