# Supplementary material for: Decoupled contrastive multi-view clustering with adaptive false negative elimination for cancer subtyping
Source: PLoS Comput Biol. 2025 Dec 4;21(12):e1013780. doi: 10.1371/journal.pcbi.1013780 (PMC12711033; doi:10.1371/journal.pcbi.1013780)
Supplement: S1 Table — (PDF) [file pcbi.1013780.s001.pdf]

S1 Table. The detailed information of the ten benchmark TCGA datasets evaluated in this work.

Datasets	Gene expression platform	Methylation platform	miRNA expression platform
AML	RNAseqv2 level 3 RSEM genes normalized	Illumina-450k level 3	Illumina mirnaseq level 3 miR gene expression
BRCA	RNAseqv2 level 3 RSEM genes normalized	Illumina-450k level 3	Illumina mirnaseq level 3 miR gene expression
COAD	RNAseqv2 level 3 RSEM genes normalized	Illumina-450k level 3	Illumina mirnaseq level 3 miR gene expression
GBM	HT-HG-U133A	Illumina-27k	UNC-miRNA-8x15K
KIRC	RNAseqv2 level 3 RSEM genes normalized	Illumina-450k level 3	Illumina mirnaseq level 3 miR gene expression
LIHC	RNAseqv2 level 3 RSEM genes normalized	Illumina-450k level 3	Illumina mirnaseq level 3 miR gene expression
LUSC	RNAseqv2 level 3 RSEM genes normalized	Illumina-450k level 3	Illumina mirnaseq level 3 miR gene expression
SKCM	RNAseqv2 level 3 RSEM genes normalized	Illumina-450k level 3	Illumina mirnaseq level 3 miR gene expression
OV	RNAseqv2 level 3 RSEM genes normalized	Illumina-27k level 3	Illumina mirnaseq level 3 miR gene expression
SARC	RNAseqv2 level 3 RSEM genes normalized	Illumina-450k level 3	Illumina mirnaseq level 3 miR gene expression
