# Supplementary material for: Decoupled contrastive multi-view clustering with adaptive false negative elimination for cancer subtyping
Source: PLoS Comput Biol. 2025 Dec 4;21(12):e1013780. doi: 10.1371/journal.pcbi.1013780 (PMC12711033; doi:10.1371/journal.pcbi.1013780)
Supplement: S2 Table — (PDF) [file pcbi.1013780.s002.pdf]

S2 Table. Summary of the high-dimensional original multi-omics datasets.

Datasets	Samples	mRNA expression	miRNA expression	DNA methylation
AML	187	20531	705	5000
BRCA	1227	20531	1046	5000
COAD	444	20531	705	5000
GBM	571	12042	534	5000
KIRC	536	20531	1046	5000
LIHC	373	20531	1046	5000
LUSC	587	20531	1046	5000
OV	598	20531	705	5000
SARC	265	20531	1046	5000
SKCM	467	20531	1046	5000
