# Supplementary material for: Decoupled contrastive multi-view clustering with adaptive false negative elimination for cancer subtyping
Source: PLoS Comput Biol. 2025 Dec 4;21(12):e1013780. doi: 10.1371/journal.pcbi.1013780 (PMC12711033; doi:10.1371/journal.pcbi.1013780)
Supplement: S3 Table — ✓ denotes that the dataset includes the clinical labels, whereas × represents that the clinical labels are absent. (PDF) [file pcbi.1013780.s003.pdf]

S3 Table. Clinical labels selected for each cancer dataset. √ denotes that the dataset includes the clinical labels, whereas × represents that the clinical labels are absent.

Datasets	age†	gender	Pathologic_T	Pathologic_M	Pathologic_N	Pathologic _stage	total number
AML	√	√	×	×	×	×	2
BRCA	√	√	√	√	√	√	6
COAD	√	√	√	√	√	√	6
GBM	√	√	×	×	×	×	2
KIRC	√	√	√	√	√	√	6
LIHC	√	√	√	√	√	√	6
LUSC	√	√	√	√	√	√	6
OV	√	√	×	×	×	×	2
SARC	√	√	×	×	×	×	2
SKCM	√	√	√	√	√	√	6

† denotes "age at initial pathologic diagnosis"
