# Supplementary material for: Decoupled contrastive multi-view clustering with adaptive false negative elimination for cancer subtyping
Source: PLoS Comput Biol. 2025 Dec 4;21(12):e1013780. doi: 10.1371/journal.pcbi.1013780 (PMC12711033; doi:10.1371/journal.pcbi.1013780)
Supplement: S4 Table — The bold numbers in the table denotes the optimal number of clusters selected for each dataset. In the A/B format, A represents the −log10 P-values of survival analysis, while B indicates the number of enriched clinical parameters. (PDF) [file pcbi.1013780.s004.pdf]

S4 Table. DCMC is evaluated across ten cancer datasets using multiple clustering configurations (3, 4, and 5 clusters). The **bold numbers** in the table denotes the optimal number of clusters selected for each dataset. In the *A/B* format, *A* represents the −log10 P-value of survival analysis, while *B* indicates the number of enriched clinical parameters.

Cluster	AML	BRCA	COAD	GBM	KIRC	LIHC	LUSC	OV	SARC	SKCM
C-3	**1/7.0**	1/4.0	1/2.5	2/7.0	3/4.7	1/5.1	**1/3.3**	1/2.5	1/4.9	1/8.7
C-4	1/4.7	2/3.8	**2/2.9**	1/5.3	**5/7.2**	2/5.6	1/2.5	1/3.1	1/6.0	1/7.5
C-5	1/6.2	**4/8.1**	1/3.2	**2/7.1**	3/6.1	**3/9.4**	1/2.4	**1/3.2**	**2/9.2**	**3/9.8**
