# Supplementary material for: Decoupled contrastive multi-view clustering with adaptive false negative elimination for cancer subtyping
Source: PLoS Comput Biol. 2025 Dec 4;21(12):e1013780. doi: 10.1371/journal.pcbi.1013780 (PMC12711033; doi:10.1371/journal.pcbi.1013780)
Supplement: S5 Table — (PDF) [file pcbi.1013780.s005.pdf]

S5 Table. The significant clinical parameters enriched by different methods.

Methods/Datasets	AML	BRCA	COAD	GBM	KIRC	LIHC	LUSC	OV	SARC	SKCM
K-means	A	A	A	A,G	A,G,M,S	A,G	A	A	A,G	A,T,M
Spectral	A	A,G	A	A,G	A,G,T,M,S	A,G	A,G	A	A,G	A,T
LRAcluster	A	A,G,T,N	A	A,G	A,G	A,G	A	A	A,G	A,T,N
CC	A	A,G,N	A	A,G	A,G,T,S	A,G,T	A	A	A,G	A
PINSPLUS	A	A,N	A	A	G,M,T	A,G	A,N	A	A,G	A
SNF	A	A,N	A,T,N	A	A,G,T,N,S	A,G	A	A	A,G	A,T
SNFCC	A	A,T	A,G	A,G	A,G,T,M,S	A,G	A	A	A,G	A,T
rMKL-LPP	A	A,T,M,N,	A,N	A,G	A,G,N,S	A,G,T,M	A	A	A,G	A,T
MCCA	A,G	A,T	A,G	A,G	M,T	A,G	A,N	A	A,G	A,T
MultiNMF	A	A	A	A	A,G,T,S	A,G,T	A	A	A,G	A,T
iClusterBayes	A	A,N	A	A,G	A,G,M,S	A,G	A	A	A,G	A,T,M,N
NEMO	A	A,G,T	A	A,G	A,G,T,M,S	A,G,M,N	A	A	A,G	A,T
DLSF	A	A,T	A	A,G	A,G,T	A,G,T	A	A	A,G	A,T,S
MSNE	A	A	A,T,M	A	M,T	A,G,M	A	A	A	A,G
DSIR	A	A,N,S	A	A,G	G,T,M,S	A,G	A,G	A	A,G	A,T,S
MRGCN	A	A,N,T,S	S	A,G	G,M,T,S	A,G	A	A	A,G	A,T,S
MOCSS	A	A,T,N	A,G	A,G	A,G,T,S	A,G	A,G,N	A	A,G	A,T,M
DMCL	A	A,T,M,N	A	A,G	A,G,T,M,S	A,G,T	A	A	A,G	A,T
DILCR	A	A,T,N	A	A,G	A,G,T,N,S	A,G	A	A	A,G	A
DCMC	A	A,G,T,N	A,G	A,G	A,G,T,M,S	A,G,N	A	A	A,G	A,T,N
