# Supplementary material for: Decoupled contrastive multi-view clustering with adaptive false negative elimination for cancer subtyping
Source: PLoS Comput Biol. 2025 Dec 4;21(12):e1013780. doi: 10.1371/journal.pcbi.1013780 (PMC12711033; doi:10.1371/journal.pcbi.1013780)
Supplement: S6 Table — During preprocessing, we exclude non-tumor samples and remove records with missing or duplicate entries. The average missing rate represents the proportion of samples with at least one missing omics modality among all retained samples. (PDF) [file pcbi.1013780.s006.pdf]

S6 Table. Summary of missing omics data across ten TCGA cancer datasets. During preprocessing, we exclude non-tumor samples and remove records with missing or duplicate entries. The average missing rate represents the proportion of samples with at least one missing omics modality among all retained samples.

**Datasets**	**Samples**	**Missing mRNA**	**Missing miRNA**	**Missing DNA methylation**	**Missing all omics**	**Average missing rate**
AML	187	25	11	6	3	7.49%
BRCA	1227	3	341	314	1	17.88%
COAD	444	167	230	157	149	41.59%
GBM	571	48	10	291	0	20.37%
KIRC	536	4	283	218	3	31.41%
LIHC	373	6	5	0	0	0.98%
LUSC	587	0	157	131	0	16.35%
OV	598	292	139	14	5	24.80%
SARC	265	2	2	0	0	0.50%
SKCM	467	6	28	5	4	2.78%
