# Supplementary material for: Decoupled contrastive multi-view clustering with adaptive false negative elimination for cancer subtyping
Source: PLoS Comput Biol. 2025 Dec 4;21(12):e1013780. doi: 10.1371/journal.pcbi.1013780 (PMC12711033; doi:10.1371/journal.pcbi.1013780)
Supplement: S7 Table — (PDF) [file pcbi.1013780.s007.pdf]

S7 Table. Comparison of clustering and survival analysis performance under different feature selection strategies across ten TCGA cancer datasets.

Datasets	Method	-log10 (P-value)	Enriched Labels	Silhouette Score	PAC Score
**AML**	Original	4.7	**2**	0.1826 ●	0.3967
	Top_2000	**7.0**	1	**0.3844**	**0.0420**
	Top_3000	5.6 ●	**2**	0.1687	0.1081 ●
	Random_3000	3.1	1	0.0287	0.7903
**BRCA**	Original	4.0	3	0.1902	**0.2504**
	Top_2000	**8.1**	**4**	**0.2120**	0.2857
	Top_3000	6.1 ●	**4**	0.1999 ●	0.2533 ●
	Random_3000	3.2	**4**	0.0687	0.3916
**COAD**	Original	2.8 ●	1	0.1261	0.4355
	Top_2000	**2.9**	**2**	**0.3450**	**0.1030**
	Top_3000	1.7	**2**	0.1907 ●	0.3253 ●
	Random_3000	1.2	1	0.1173	0.4045
**GBM**	Original	6.4	**2**	0.1683	0.1653 ●
	Top_2000	7.1 ●	**2**	**0.2796**	**0.1231**
	Top_3000	**7.4**	**2**	0.1974 ●	0.1839
	Random_3000	3.1	1	0.0486	0.5363
**KIRC**	Original	6.2 ●	**5**	0.2904 ●	0.2589
	Top_2000	**7.2**	**5**	**0.3853**	**0.1452**
	Top_3000	5.3	**5**	0.1956	0.1642 ●
	Random_3000	4.3	3	0.0656	0.5644
**LIHC**	Original	8.0 ●	3	0.2027	0.2294
	Top_2000	**9.4**	3	**0.3684**	**0.1513**
	Top_3000	8.4 ●	3	0.2731 ●	0.1787 ●
	Random_3000	4.0	3	0.0518	0.3003
**LUSC**	Original	2.5	**2**	0.2414 ●	0.1548 ●
	Top_2000	**3.3**	1	**0.3154**	**0.1438**
	Top_3000	2.9 ●	**2**	0.1687	0.2769
	Random_3000	1.5	1	0.0722	0.4070
**OV**	Original	3.0 ●	1	0.2377 ●	0.3074
	Top_2000	**3.2**	1	**0.3154**	0.2917 ●
	Top_3000	2.3	1	0.1421	**0.2848**
	Random_3000	1.5	1	0.0997	0.3822
**SARC**	Original	8.7 ●	2	0.1835	0.2136 ●
	Top_2000	**9.2**	2	**0.3694**	**0.0859**
	Top_3000	8.0	2	0.2087 ●	0.2426
	Random_3000	5.2	2	0.0859	0.4798
**SKCM**	Original	6.5	2	0.2090	0.2451
	Top_2000	**9.8**	**3**	**0.3341**	**0.1788**
	Top_3000	7.4 ●	2	0.2298 ●	0.2429 ●
	Random_3000	4.9	2	0.0959	0.4493

Note: The Silhouette Score represents intra-cluster cohesion and inter-cluster separation, where **a higher value** indicates better clustering quality. The PAC score quantifies clustering stability, and **a lower value** reflects more consistent results across subsamples. The best results in each dataset are shown in **bold** face, and • indicates the second-best result.
