# Supplementary material for: Decoupled contrastive multi-view clustering with adaptive false negative elimination for cancer subtyping
Source: PLoS Comput Biol. 2025 Dec 4;21(12):e1013780. doi: 10.1371/journal.pcbi.1013780 (PMC12711033; doi:10.1371/journal.pcbi.1013780)
Supplement: S8 Table — (PDF) [file pcbi.1013780.s008.pdf]

S8 Table. Comparison between shared and specific decoders across ten cancer datasets.

Datasets	Shared Decoders				Specific Decoders			
	-log10 (P-values)	Enriched Clinical Labels	Silhouette Score	PAC Score	-log10 (P-values)	Enriched Clinical Labels	Silhouette Score	PAC
AML	4.2	1	0.0921	0.5103	**7.0**	1	**0.3844**	**0.0420**
BRCA	4.4	3	0.0541	0.3937	**8.1**	**4**	**0.2120**	**0.2857**
COAD	1.3	2	0.0821	0.3929	**2.9**	2	**0.3450**	**0.1030**
GBM	5.4	2	0.1192	0.3699	**7.1**	2	**0.2796**	**0.1231**
KIRC	6.1	5	0.0791	0.4674	**7.2**	5	**0.3853**	**0.1452**
LIHC	7.2	3	0.1016	0.2142	**9.4**	3	**0.3684**	**0.1513**
LUSC	1.8	1	0.0487	0.5850	**3.3**	1	**0.3154**	**0.1438**
OV	1.6	1	0.0762	**0.2164**	**3.2**	1	**0.3154**	0.2917
SARC	6.3	2	0.1324	0.2212	**9.2**	2	**0.3694**	**0.0859**
SKCM	8.0	2	0.0620	0.3496	**9.8**	**3**	**0.3341**	**0.1788**

Note: The Silhouette Coefficient represents intra-cluster cohesion and inter-cluster separation, where a higher value indicates better clustering quality. The PAC score quantifies clustering stability, and a lower value reflects more consistent results across subsamples. The best results are shown in **bold** face.
