# Supplementary material for: Decoupled contrastive multi-view clustering with adaptive false negative elimination for cancer subtyping
Source: PLoS Comput Biol. 2025 Dec 4;21(12):e1013780. doi: 10.1371/journal.pcbi.1013780 (PMC12711033; doi:10.1371/journal.pcbi.1013780)
Supplement: S9 Table — Note: The best results in each dataset are shown in bold face, and • indicates the second-best result. (PDF) [file pcbi.1013780.s009.pdf]

S9 Table. Sensitivity analysis of decoder architecture on AML and LIHC datasets. Note: The best results in each dataset are shown in **bold** face, and • indicates the second-best result.

Datasets	Configuration	-log10 (P-values)	Enriched Clinical Labels	Silhouette Score	PAC Score
AML	depth=3	6.5 •	2	0.1180	0.3398
	depth=5	5.2	2	0.2143 •	0.2027 •
	Default Settings	**7.0**	1	**0.3844**	**0.0420**
	width_mult=0.5	5.5	1	0.1710	0.2243
	width_mult=2.0	4.7	2	0.1557	0.2122
LIHC	depth=3	6.6	4	0.0924	0.2779
	depth=5	7.7 •	3	0.1401 •	0.1960 •
	Default Settings	**9.4**	3	**0.3684**	**0.1513**
	width_mult=0.5	6.7	3	0.1064	0.2303
	width_mult=2.0	6.9	3	0.1108	0.2895
