# Supplementary material for: Decoupled contrastive multi-view clustering with adaptive false negative elimination for cancer subtyping
Source: PLoS Comput Biol. 2025 Dec 4;21(12):e1013780. doi: 10.1371/journal.pcbi.1013780 (PMC12711033; doi:10.1371/journal.pcbi.1013780)
Supplement: S10 Table — Each cell presents the results in the format A/B(C), where A denotes the number of enriched clinical labels, B is the −log10 P-values from survival analysis, and C indicates the number of clusters. Means represent the algorithm’s average value. Bold values highlights the superior results obtained by methods integrated with AFNE. (PDF) [file pcbi.1013780.s010.pdf]

S10 Table. Performance comparison of competitive methods with and without Adaptive False Negative Elimination (AFNE) across ten TCGA cancer datasets. Each cell presents the results in the format $A/B(C)$, where A denotes the number of enriched clinical labels, B is the $-\log 10\, P$-values from survival analysis, and C indicates the number of clusters. Means represent the algorithm's average value. **Bold values** highlight the superior results obtained by methods integrated with AFNE.

**Methods**	**AML**	**BRCA**	**COAD**	**GBM**	**KIRC**	**LIHC**	**LUSC**	**OV**	**SARC**	**SKCM**	**Means**
DLSF	1/2.5(5)	2/1.9(3)	1/0.1(4)	2/4.5(5)	3/2.8(4)	3/3.3(3)	1/0.1(3)	1/0.3(4)	2/2.4(10)	3/3.9(5)	1.9/2.2
DLSF+AFNE	1/**2.9**(5)	2/**2.2**(3)	1/0.1(4)	2/3.4(5)	4/1.8(4)	2/**3.7**(3)	1/**0.2**(3)	**2/0.5**(4)	2/**3.7**(10)	2/**5.1**(5)	1.9/**2.4**
MOCSS	1/3.5(4)	3/2.8(5)	2/0.8(5)	2/5.2(3)	4/4.0(4)	2/0.7(3)	3/0.4(5)	1/0.6(3)	2/2.2(4)	3/5.0(5)	2.3/2.5
MOCSS+AFNE	1/**4.0**(4)	3/**3.9**(5)	2/**1.0**(5)	2/**5.9**(3)	**5/4.2**(4)	2/**1.7**(3)	2/**0.6**(5)	1/**0.7**(3)	2/**2.7**(4)	3/**5.1**(5)	2.3/**3.0**
DMCL	1/2.1(9)	4/2.1(3)	1/0.1(2)	2/0.1(4)	5/1.7(6)	2/2.6(4)	1/0.2(4)	1/0.7(5)	2/3.2(6)	2/2.3(5)	2.1/1.5
DMCL+AFNE	1/**2.4**(9)	4/**3.1**(3)	1/**0.7**(2)	2/**1.5**(4)	5/**1.9**(6)	2/2.6(4)	1/**0.4**(4)	1/**0.8**(5)	2/**3.7**(6)	2/**2.6**(5)	2.1/**2.0**
DILCR	1/5.5(5)	3/3.1(5)	1/0.8(3)	2/2.5(5)	5/1.6(4)	2/2.9(4)	1/0.6(3)	1/0.6(2)	2/2.1(5)	1/1.1(5)	1.9/2.1
DILCR+AFNE	1/**6.1**(5)	3/**3.6**(5)	1/**1.0**(3)	2/**4.1**(5)	5/**3.0**(4)	2/**3.1**(4)	1/**1.1**(3)	1/**1.6**(2)	2/**3.4**(5)	**3/2.9**(5)	**2.1/3.0**
DCMC(ours)	1/7.0(3)	4/8.1(4)	2/2.9(4)	2/7.1(5)	5/7.2(4)	3/9.4(5)	1/3.3(3)	1/3.2(5)	2/9.2(5)	3/9.8(5)	2.4/6.7
