# Supplementary material for: Decoupled contrastive multi-view clustering with adaptive false negative elimination for cancer subtyping
Source: PLoS Comput Biol. 2025 Dec 4;21(12):e1013780. doi: 10.1371/journal.pcbi.1013780 (PMC12711033; doi:10.1371/journal.pcbi.1013780)
Supplement: S11 Table — In each cell, results are shown as A/B, where A represents the number of significant clinical labels and B denotes the −log10 P-values obtained from survival analysis. (PDF) [file pcbi.1013780.s011.pdf]

S11 Table. Clustering performance of DCMC across the ten cancer datasets under different view combinations. In each cell, results are shown as *A/B*, where *A* represents the number of significant clinical labels and *B* denotes the −log10 P-values obtained from survival analysis.

Cluster	AML	BRCA	COAD	GBM	KIRC	LIHC	LUSC	OV	SARC	SKCM
exp+augmentation	1/4.2	1/3.3	1/2.7	1/3.6	3/4.5	2/5.4	1/2.0	1/3.1	2/3.5	1/6.5
mirna+augmentation	1/6.7	1/3.2	1/2.6	1/2.6	4/5.6	2/5.8	1/2.4	1/2.4	2/4.2	2/3.7
methy+augmentation	1/5.9	1/3.0	2/1.7	2/6.0	4/5.6	2/4.0	1/1.3	1/1.3	1/7.2	1/5.5
exp+mirna	1/6.0	1/4.5	1/2.3	1/5.0	2/6.0	2/7.2	1/2.8	1/2.5	2/4.8	2/9.3
exp+methy	1/5.2	1/5.3	1/2.8	1/5.3	4/5.7	2/7.0	1/1.7	1/2.1	1/5.8	2/7.9
mirna+methy	1/5.4	1/4.5	1/2.3	1/7.0	3/5.5	1/4.8	1/2.9	1/2.5	2/5.0	2/6.2
exp+mirna+methy	1/7.0	4/8.1	2/2.9	2/7.1	5/7.2	3/9.4	1/3.3	1/3.2	1/9.2	2/9.8
