# Supplementary material for: Decoupled contrastive multi-view clustering with adaptive false negative elimination for cancer subtyping
Source: PLoS Comput Biol. 2025 Dec 4;21(12):e1013780. doi: 10.1371/journal.pcbi.1013780 (PMC12711033; doi:10.1371/journal.pcbi.1013780)
Supplement: S12 Table — (PDF) [file pcbi.1013780.s012.pdf]

S12 Table. Memory usage (MB) of DCMC and baseline methods across 10 TCGA datasets.

Methods	AML	BRCA	COAD	GBM	KIRC	LIHC	LUSC	OV	SARC	SKCM
K-means	436	1628	551	684	487	958	895	715	672	1187
Spectral	41	260	57	76	46	119	107	82	72	160
LRAcluster	4115	18103	5694	6611	4661	10020	9354	7051	6979	13030
PINPLUS	286	358	278	285	273	303	298	288	285	319
SNF	200	2379	324	489	231	860	747	535	436	1263
SNFCC	2965	37320	4908	7495	3443	13252	11505	8226	6574	19616
MCCA	4668	16698	5113	5552	4894	10087	9229	6686	6295	9173
iClusterBayes	471	1796	611	744	531	1060	991	793	746	1303
NEMO	70	653	109	150	81	255	228	168	141	360
DLSF	224	362	248	247	262	297	295	258	281	319
MOCSS	55	171	71	88	61	125	117	94	87	153
DMCL	296	378	330	369	330	373	371	371	344	370
DILCR	365	402	369	367	377	385	386	369	381	393
DCMC(ours)	291	325	295	293	299	306	305	298	303	309
