# Supplementary material for: Decoupled contrastive multi-view clustering with adaptive false negative elimination for cancer subtyping
Source: PLoS Comput Biol. 2025 Dec 4;21(12):e1013780. doi: 10.1371/journal.pcbi.1013780 (PMC12711033; doi:10.1371/journal.pcbi.1013780)
Supplement: S13 Table — (PDF) [file pcbi.1013780.s013.pdf]

S13 Table. Run time comparisons of baseline methods on all datasets(in seconds).

Methods	AML	BRCA	COAD	GBM	KIRC	LIHC	LUSC	OV	SARC	SKCM
K-means	1.22	10.4	2.51	4.01	2.53	2.97	2.96	3.06	3.30	3.28
Spectral	0.06	1.22	0.16	0.18	0.09	0.38	0.33	0.19	0.25	0.66
LRAcluster	4.55	65.46	7.02	10.91	5.33	21.07	17.48	12.25	10.45	33.15
PINPLUS	11.91	148.23	29.94	10.93	13.63	61.83	28.53	31.75	31.01	78.75
SNF	9.08	82.66	17.83	25.54	26.15	27.03	30.51	25.23	15.54	36.67
SNFCC	4.22	87.34	7.36	11.85	5.17	23.15	19.36	13.04	9.96	37.34
MCCA	4.87	22.13	4.80	7.15	5.55	11.82	10.60	7.49	6.57	10.15
iClusterBayes	425.27	1800.17	600.66	661.29	477.77	907.59	874.49	707.12	651.84	1138.75
NEMO	8.41	62.65	16.34	24.75	27.21	22.33	28.37	29.72	15.55	29.43
DLSF	19.79	30.31	20.40	23.03	20.95	21.90	22.23	21.83	21.43	24.43
MOCSS	107.69	341.94	96.33	158.58	104.12	209.82	147.44	106.23	101.01	164.20
DMCL	19.09	170.04	11.97	34.21	14.19	37.39	46.61	41.78	10.60	53.72
DILCR	35.32	214.61	70.95	76.57	49.97	176.58	139.11	79.73	65.94	146.28
DCMC(ours)	2099	23840	7860	9980	10157	9680	11310	7977	5047	11334
