# Supplementary material for: Decoupled contrastive multi-view clustering with adaptive false negative elimination for cancer subtyping
Source: PLoS Comput Biol. 2025 Dec 4;21(12):e1013780. doi: 10.1371/journal.pcbi.1013780 (PMC12711033; doi:10.1371/journal.pcbi.1013780)
Supplement: S14 Table — (PDF) [file pcbi.1013780.s014.pdf]

S14 Table. Sensitivity analysis of threshold and top-k selection on survival significance across ten cancer datasets.

**Threshold/Top_k**		**AML** (P-val)	**BRCA** (P-val)	**COAD** (P-val)	**GBM** (P-val)	**KIRC** (P-val)	**LIHC** (P-val)	**LUSC** (P-val)	**OV** (P-val)	**SARC** (P-val)	**SKCM** (P-val)
Th<0.5	**K=1**	3.2	6.7	2.0	5.7	5.4	6.3	1.9	2.3	5.7	7.2
	**K=2**	2.8	5.0	2.3	5.2	6.0	6.8	2.2	3.3	6.5	6.3
	**K=3**	3.3	3.8	2.3	4.8	5.8	6.8	2.1	2.5	5.9	8.3
	**K=4**	3.6	5.2	2.0	5.9	5.4	7.0	2.0	2.8	8.2	7.3
Th<0.6	**K=1**	4.2	4.2	2.2	4.5	5.4	6.6	2.6	3.1	5.1	7.5
	**K=2**	4.7	4.1	2.0	4.7	5.4	7.4	3.0	3.0	6.0	9.0
	**K=3**	4.9	3.9	2.3	6.3	5.6	7.6	2.2	3.1	6.0	7.5
	**K=4**	4.6	4.5	2.6	5.0	5.6	6.3	2.4	2.2	5.0	7.9
Th<0.7	**K=1**	5.8	5.2	2.8	4.9	6.8	7.0	2.1	2.6	6.3	8.2
	**K=2**	6.4	5.2	**3.2**	5.5	5.4	9.0	3.0	2.6	5.7	7.4
	**K=3**	**7.0**	**8.1**	2.9	**7.2**	**7.2**	**9.4**	**3.3**	**3.2**	**9.2**	**9.8**
	**K=4**	5.6	4.2	2.8	6.1	6.0	6.7	2.4	**3.2**	6.3	6.5
Th<0.8	**K=1**	4.8	5.8	2.2	5.1	5.5	7.4	2.0	3.3	5.4	9.7
	**K=2**	5.5	4.8	2.4	5.1	5.3	7.0	1.9	2.7	7.7	7.6
	**K=3**	5.8	5.5	2.9	5.1	5.5	6.9	2.0	**3.2**	5.2	6.1
	**K=4**	4.9	4.7	2.6	5.4	5.3	6.4	1.8	3.1	6.0	7.1
Th<0.9	**K=1**	4.7	4.6	2.4	5.0	5.5	7.4	2.1	3.7	6.3	7.2
	**K=2**	5.2	5.0	2.2	5.0	5.3	6.6	2.5	2.7	5.7	6.7
	**K=3**	5.3	4.1	2.2	5.0	5.5	6.3	2.6	2.5	7.1	7.0
	**K=4**	4.6	5.1	2.4	6.5	5.3	7.5	2.3	2.6	6.0	6.9

Note: The best result for each dataset (column) is shown in **bold** face. P-val represents the $-\log 10\ P$-values obtained from survival analysis.
