# Supplementary material for: Decoupled contrastive multi-view clustering with adaptive false negative elimination for cancer subtyping
Source: PLoS Comput Biol. 2025 Dec 4;21(12):e1013780. doi: 10.1371/journal.pcbi.1013780 (PMC12711033; doi:10.1371/journal.pcbi.1013780)
Supplement: S1 Text — (DOCX) [file pcbi.1013780.s020.docx]

**S1 Text**. **The description of pan-cancer dataset.**

To evaluate the model’s performance on a large and heterogeneous cohort, we construct a pan-cancer dataset by integrating multi-omics data from eight distinct cancer types available from The Cancer Genome Atlas (TCGA)[1]. The cancer types included are Acute Myeloid Leukemia (AML), Breast Invasive Carcinoma (BRCA), Colon Adenocarcinoma (COAD), Glioblastoma Multiforme (GBM), Kidney Renal Clear Cell Carcinoma (KIRC), Liver Hepatocellular Carcinoma (LIHC), Lung Squamous Cell Carcinoma (LUSC), Ovarian Serous Cystadenocarcinoma (OV), Sarcoma (SARC), and Skin Cutaneous Melanoma (SKCM).

Following a similar protocol to previous studies[2], we exclude the GBM and OV datasets, as their DNA methylation data were generated on a different platform. For each of the eight selected cancer types, we preprocess each omics dataset by removing miRNAs with zero variance and selecting the top 2000 genes and CpG loci with the highest variance for downstream analysis. Each omics dataset is then normalized to have zero mean and unit variance. As a result, the final pan-cancer cohort consists of 2607 samples. Each sample is characterized by a profile of 2000 gene expression features, 2000 CpG methylation loci, and 516 miRNA expression features. The cancer type of origin for each sample serves as its ground-truth label for our analysis.

1. Rappoport N, Shamir R. Multi-omic and multi-view clustering algorithms: review and cancer benchmark. Nucleic Acids Research. 2018;46(20):10546–1062. https://doi.org/10.1093/nar/gky889. PMID: 30295871.
2. Chen W, Wang H, Liang C. Deep multi-view contrastive learning for cancer subtype identification. Briefings in Bioinformatics. 2023;24(5):bbad282. https://doi.org/10.1093/bib/bbad282. PMID: 37539822.
